# YhbO is a DJ-1 family glyoxalase and α-oxoaldehyde hydratase that confers resistance to reactive carbonyl stress

**DOI:** 10.1016/j.jbc.2026.113300

**Published:** 2026-06-29

**Authors:** Aiko Watanabe, Kai Kanematsu, Yohei Hizukuri, Tatsuki Hori, Shizuka Ogiwara, Tomohiko Okuno, Mirei Saito, Tomoya Ito, Chihiro Shibuya, Yoshinori Akiyama, Shinji Toyota, Yoshitaka Moriwaki, Koji Yamano, Fumika Koyano, Takashi Kajitani, Ryuichiro Ishitani, Masahiro Yamashina, Hidetaka Kosako, Noriyuki Matsuda

**Affiliations:** 1 Department of Biomolecular Pathogenesis, Division of Advanced Pathophysiological Science, Medical Research Laboratory, Institute of Integrated Research, Institute of Science Tokyo, Tokyo, Japan; 2 Laboratory of Biological Membrane System, Institute for Life and Medical Sciences, Kyoto University, Sakyo-ku, Kyoto, Japan; 3 Department of Computational Drug Discovery and Design, Division of Biological Data Science, Medical Research Laboratory, Institute of Integrated Research, Institute of Science Tokyo, Tokyo, Japan; 4 Department of Chemistry, School of Science, Institute of Science Tokyo, Tokyo, Japan; 5 Intracellular Quality Control Project, Tokyo Metropolitan Institute of Medical Science, Tokyo, Japan; 6 Core Facility Center, Research Infrastructure Management Center, Institute of Science Tokyo, Yokohama, Japan; 7 Division of Cell Signaling, Institute of Advanced Medical Sciences, Tokushima University, Tokushima, Japan

**Keywords:** Parkinson disease, PARK7, enzyme catalysis, enzyme mechanism, hydrolase, DJ-1, oxo-aldehyde, prokaryote, hydratase, catalytic triad

## Abstract

Reactive α-oxoaldehydes, such as glyoxal (GO) and methylglyoxal (MGO), are cytotoxic glycolysis byproducts that induce cellular dysfunction by nonenzymatically modifying diverse macromolecules. Although DJ-1 (PARK7), a protein linked to Parkinson’s disease, acts as a type-III glyoxalase/methylglyoxalase, the physiological function(s) of its prokaryotic homologs remain poorly understood. Here, we investigated the enzymatic and physiological properties of YhbO, one of four DJ-1 homologs in *Escherichia coli*. We demonstrate that YhbO has higher catalytic efficiency than DJ-1 and exhibits broad-spectrum α-oxoaldehyde hydratase activity against GO, MGO, and phenylglyoxal. Structural modeling and mutational analyses revealed that residues E17, G73, G74, C104, and H105 are important for catalysis, whereas D78, previously thought to comprise a Cys–His–Asp triad, is dispensable. Notably, H105 is strictly required, distinguishing the YhbO catalytic mechanism from that of DJ-1 and HchA. Further underscoring mechanistic divergence within the DJ-1 family, YhbO converted MGO into both L- and D-lactate, whereas DJ-1 generates only L-lactate. In cell-based assays, overexpression of wild-type YhbO conferred resistance to GO- and MGO-induced stress and suppressed the formation of GO- and MGO-derived adducts such as carboxymethyl-lysine and methylglyoxal-hydroimidazolone. In contrast, catalytically impaired YhbO mutants were unable to suppress adduct formation. Further, comparable levels of endogenous GO- or MGO-derived adducts in cells with YhbO knocked out, or a YhbO/HchA double knockout, suggest redundancy within the canonical glyoxalase I pathway. Taken together, these findings establish YhbO as a unique α-oxoaldehyde hydratase with distinct catalytic requirements and provide insights into the mechanistic diversity within the DJ-1 protein family.

Reactive α-oxoaldehydes such as glyoxal (GO) and methylglyoxal (MGO) are highly cytotoxic metabolic byproducts generated endogenously during glycolysis (1, 2, 3, 4). These reactive carbonyl species non-enzymatically modify proteins, nucleic acids, and other biomolecules, resulting in the formation of advanced glycation end-products (AGEs) that contribute to cellular dysfunction, aging, and disease pathogenesis. Therefore, efficient detoxification of α-oxoaldehydes is thought to be essential for preserving cellular function and preventing disease progression.

DJ-1 (also known as *PARK7* gene product) is a multifunctional protein linked to autosomal recessive Parkinson’s disease (5). It has been reported that DJ-1 acts as a glyoxalase III enzyme that converts reactive α-oxoaldehydes, such as GO and MGO, into less toxic α-hydroxy acids (6, 7, 8). In addition to its α-oxoaldehyde detoxification activity, DJ-1 has been implicated in cellular defense against oxidative and mitochondrial stress (9, 10, 11). Although other diverse molecular functions, such as molecular chaperone activity (12, 13), transcriptional regulation (14, 15), and protease activity (16) have also been attributed to DJ-1, their relevance remains debated (17). More recently, other research groups and we demonstrated that DJ-1 prevents the formation of phosphoglycerate adducts derived from the reactive glycolytic intermediate cyclic 3-phosphoglyceric anhydride (cPGA) (18, 19, 20). Based on these findings, we believe that the genuine function of DJ-1 is hydrolytic elimination of reactive metabolites derived from glycolysis. Despite these insights, the physiological functions of other DJ-1 family proteins remain to be elucidated.

DJ-1 belongs to the highly conserved PfpI/Hsp31/DJ-1 superfamily of proteins that are characterized by a nucleophile elbow motif containing a conserved Cys that is reminiscent of the catalytic center in hydrolases such as cysteine proteases (21, 22, 23, 24). In *Escherichia coli*, four DJ-1 homologs have been identified: YajL, YhbO, HchA, and ElbB (25). Our recent evolutionary analysis of DJ-1 and its four prokaryotic homologs revealed that YajL, the DJ-1 counterpart in *E*. *coli*, possesses cPGA hydrolase activity, whereas the other three homologs do not (20). While YhbO exhibits clear glyoxalase and methylglyoxalase activities (26), the YhbO catalytic mechanism and the identity of the residues essential for the hydratase activity have not been determined. Moreover, the kinetics of the enzyme's potential activity against other reactive carbonyl species, such as phenylglyoxal (PGO), have not been well defined, and it is unknown if the protective effects of YhbO against carbonyl stress in *E. coli* (26) require enzymatic activity.

To clarify the enzymatic properties of YhbO and establish its physiological significance, comprehensive biochemical and cell-based analyses were performed. Our findings have revealed that YhbO possesses broad-spectrum α-oxoaldehyde hydratase activity, which surpasses that of DJ-1, and that the enzyme provides cytoprotection against GO in the medium and suppresses AGE accumulation when overproduced *in vivo*. Additionally, by examining how various mutations impacted YhbO cytoprotection, we identified amino acid residues critical for its glyoxalase activity both *in vitro* and *in vivo*. Moreover, by combining mutational analysis with structural simulations, we demonstrated that the YhbO enzymatic reaction mechanism differs from that used by DJ-1 and HchA, even though all of the DJ-1 family proteins share similar α-oxoaldehyde hydratase activity.

## Results

### YhbO exhibits hydratase activity toward a broad range of α-oxoaldehydes

We previously demonstrated that DJ-1 possesses cPGA hydrolase, esterase, phenylglyoxalase, and methylglyoxalase activities, at least *in vitro* (20, 27, 28). However, among the DJ-1 enzymatic activities, cPGA hydrolase activity is currently considered to be the most physiologically relevant. As mentioned above, the genome of *E. coli* encodes four DJ-1 homologs: YajL, YhbO, HchA, and ElbB. Interestingly, the enzymatic activities retained by these DJ-1 homologs are distinct from one another. Although YajL, a DJ-1 counterpart of *E. coli*, possesses cPGA hydrolase activity, YajL lacks esterase activity, whereas HchA has both phenylglyoxalase and methylglyoxalase activities, but neither the cPGA hydrolase nor esterase activities (20, 28). To determine if the four DJ-1 homologs had glyoxalase and methylglyoxalase activities, Lee *et al.* used HPLC-based methods to show that YhbO and HchA have both activities, but that YajL and ElbB only possess glyoxalase activity (26). The study, however, did not utilize continuous time-course measurements, and the phenylglyoxalase activity of YhbO has yet to be tested. To overcome these limitations, we established more beginner-friendly experimental protocols (*i.e.*, spectrophotometric and fluorometric assays) that provided for real-time monitoring of enzymatic activity within a single reaction setup. Moreover, we assessed whether YhbO exhibits broad hydratase activity against various α-oxoaldehydes including phenylglyoxal (PGO).

**Figure 1:**
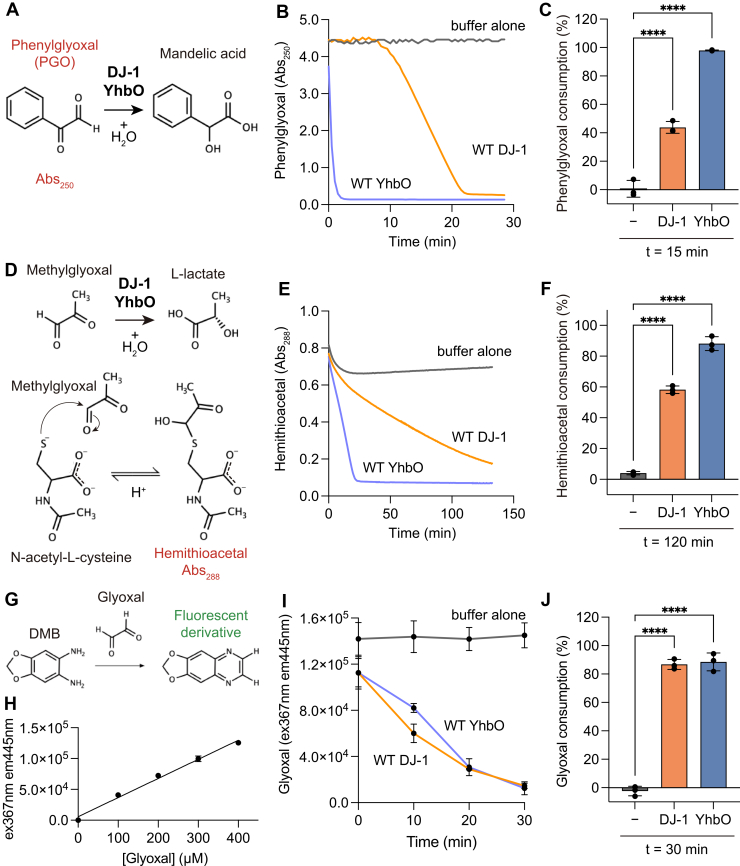
**YhbO catalyzes the conversion of α-oxoaldehydes *in vitro*.***A*, Principle of phenylglyoxal (PGO) quantification. The PGO A250 disappears when it is converted to mandelic acid by DJ-1 or YhbO. *B*, DJ-1 or YhbO (5 μM) was incubated with 1 mM PGO at 37 °C, and A250 was monitored over time. *C*, PGO consumption rate was calculated after 15 min under the same conditions as in *B* (n = 3). *D*, Methylglyoxal (MGO) quantification assay. MGO reacts with N-acetyl-L-cysteine (NAC) to form a hemithioacetal (HTA). Methylglyoxalase activity of DJ-1 or YhbO shifts the equilibrium, resulting in a reduction in A288. *E*, DJ-1 or YhbO (5 μM) was incubated with 7.5 mM MGO and 7.5 mM NAC at 37 °C, and A288 was recorded over time. *F*, the HTA consumption rate at 120 min was quantified in E (n = 3). *G*, quantification of glyoxal (GO) is based on the fluorescence of GO adducts with 1,2-diamino-4,5-methylenedioxybenzene. *H*, fluorescence spectra (Ex 367 nm, Em 445 nm) were measured under the indicated GO concentration. *I*, DJ-1 or YhbO (0.5 μM) was incubated with GO (400 μM) at 37 °C for the indicated times and then reacted with DMB for 40 min at 60 °C, and fluorescence (Ex 367 nm, Em 445 nm) was measured (n = 3). *J*, the GO consumption rate at 30 min was determined from I (n = 3). *Black*, *orange*, and *purple lines* represent buffer control, DJ-1, and YhbO, respectively (*B*, *E*, and *I*). Data in *B* and *E* are representative of three independent experiments. Data in (*C*, *F*, *H* and *J*) are the mean ± SD of three experiments (n = 3). ∗*p* < 0.05 using one-way ANOVA with Dunnett’s multiple comparisons test (*C*, *F*, *H*, *I* and *J*).

To evaluate phenylglyoxalase activity, we monitored PGO-derived absorbance at 250 nm (A250) and determined that time-dependent reduction in absorbance by DJ-1 family proteins corresponds to their conversion of PGO into mandelic acid (Fig. 1*A*) (28). Both DJ-1 and YhbO catalyzed a time-dependent reduction in absorbance. However, PGO consumption by YhbO at 15 min was nearly 100%, whereas DJ-1 was only 45% (Fig. 1, *B* and *C*). These results demonstrate that YhbO, like DJ-1, exhibits phenylglyoxalase activity, but that YhbO activity is more robust than DJ-1 (Fig. 1*B*).

We next monitored methylglyoxalase activity using a previously established assay in which MGO forms a hemithioacetal (HTA) adduct with N-acetyl-L-cysteine (NAC), which is detectable by A288 (7, 27, 28). When MGO is converted to L-lactate by DJ-1, the reaction equilibrium shifts and the absorbance decreases as illustrated in Figure 1*D*. Incubation of preformed MGO–NAC adducts with DJ-1 or YhbO resulted in a time-dependent reduction in A288 nm, with the consumption of HTA reaching ∼90% for YhbO and ∼60% for DJ-1 after 120 min (Fig. 1, *E* and *F*). These results indicate that the MGO-degrading activity of YhbO also exceeds that of DJ-1 (Fig. 1*E*).

To evaluate glyoxalase activity, we established a fluorescence-based assay using GO and the fluorescent probe DMB (1,2-diamino-4,5-methylenedioxybenzene) (29), which forms a fluorescent adduct (Ex 367 nm/Em 445 nm) when reacted with GO (Fig. 1*G*). We first verified that the GO concentration correlates with fluorescence intensity, confirming the feasibility of using this method to quantify GO (Fig. 1*H*). Incubation of GO with DJ-1 or YhbO followed by DMB addition revealed a time-dependent reduction in fluorescence, indicating enzymatic-based consumption of GO (Fig. 1, *I* and *J*). After 30 min, GO degradation reached approximately 90% for both DJ-1 and YhbO (Fig. 1*J*). Collectively, these findings demonstrate that YhbO exhibits hydratase activity for structurally diverse α-oxoaldehydes, including PGO, MGO, and GO.

### YhbO exhibits higher catalytic efficiency than DJ-1 for α-oxoaldehyde substrates

**Figure 2:**
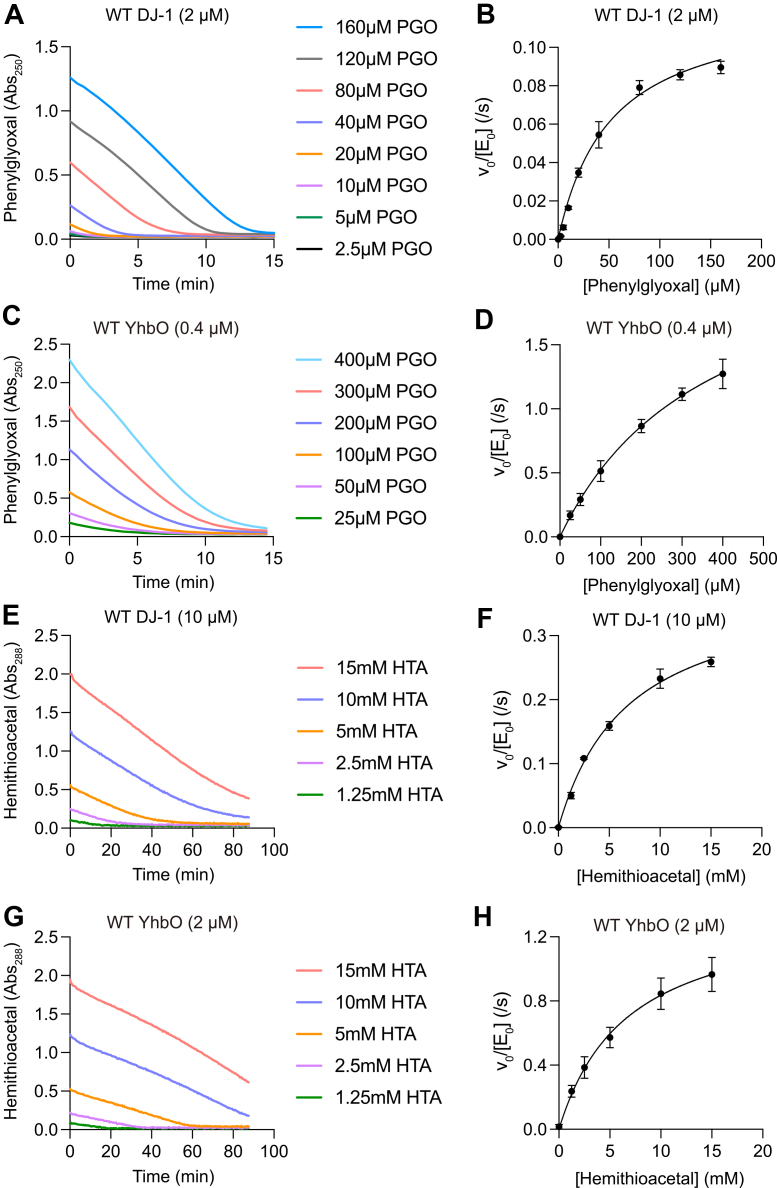
**The catalytic efficiency of YhbO with α-oxoaldehydes exceeds that of DJ-1.***A*, DJ-1 (2 μM) was incubated with PGO at the indicated concentrations, and A250 was recorded over time. *B*, initial catalytic velocities were derived from A and fitted to a Michaelis-Menten equation. *C, D*, YhbO (0.4 μM) was incubated with PGO at the indicated concentrations, and a Michaelis-Menten plot was obtained as in *A* and *B*. *E*–*H*. DJ-1 (*E*, *F*) or YhbO (*G*, *H*) was incubated with MGO-derived HTA at the indicated concentrations, and Michaelis-Menten plots were generated. Vmax and Km were calculated by curve fitting of the Michaelis-Menten, and *k*_cat_ was obtained as Vmax/[E_0_]. The results are summarized in Tables 1 and 2. Data in *A*, *C*, *E*, and *G* are representative of three independent experiments. Data in *B*, *D*, *F*, and *H* are the mean ± SD of three replicates (n = 3).

To characterize the catalytic properties of YhbO in more detail, we performed steady-state kinetic analyses using PGO and MGO as substrates. Wild-type (WT) DJ-1 and YhbO were incubated with varying concentrations of each substrate, and the progression of the reaction was monitored by spectrophotometric changes in A250 (PGO) or A288 (HTA derived from MGO and NAC) as shown in Figure 1. Initial velocities were calculated and then plotted to generate Michaelis–Menten curves. For phenylglyoxalase activity, DJ-1 was incubated with increasing concentrations of PGO, and the time-dependent decrease in A250 was recorded (Fig. 2*A*). The resulting Michaelis–Menten plot (Fig. 2*B*) of DJ-1 revealed a V_max_ of 2.49 × 10^−7^ M/s, consistent with our previous report (28). YhbO showed a similar time-dependent reduction in absorbance (Fig. 2*C*), and the corresponding Michaelis–Menten plot (Fig. 2*D*) yielded a V_max_ of 9.85 × 10^−7^ M/s, substantially higher than that of DJ-1. The kinetic parameters, derived by nonlinear curve fitting, showed that the *K*_m_ of YhbO for PGO (∼370 μM) was approximately 7-fold higher than that of DJ-1 (∼52 μM), indicating lower substrate affinity. However, YhbO had markedly higher catalytic turnover, with a *k*_cat_ of ∼2.5 s^−1^ compared to ∼0.12 s^−1^ for DJ-1. Consequently, the catalytic efficiency (*k*_cat_/*K*_m_) of YhbO (∼6700 M^−1^ s^−1^) was approximately 2.8-fold greater than that of DJ-1 (∼2400 M^−1^ s^−1^), demonstrating that YhbO has significantly higher phenylglyoxalase activity than DJ-1 (Table 1).

**Table 1:** Phenylglyoxalase kinetics of DJ-1, YhbO, and the YhbO D78A mutant

Protein	*K*_m_ (M)	*k*_cat_ (s^−1^)	*k*_cat_/*K*_m_ (s^−1^ M^−1^)
WT DJ-1	5.22 ± 0.65 × 10^−5^	0.12 ± 0.007	2396 ± 228.7
WT YhbO	3.72 ± 0.49 × 10^−4^	2.46 ± 0.096	6686 ± 818.0
YhbO (D78A)	1.49 ± 0.34 × 10^−4^	1.17 ± 0.103	8050 ± 1155

We next evaluated methylglyoxalase activity. As shown above, MGO forms an HTA adduct with NAC, which exhibits an absorption peak at 288 nm (Fig. 1*D*). The time-dependent reduction in absorbance was measured for DJ-1 (Fig. 2*E*) and YhbO (Fig. 2*G*), and initial velocities were plotted against substrate concentration to generate Michaelis–Menten curves (Fig. 2, *F* and *H*, respectively). Both enzymes displayed comparable *K*_m_ values of ∼7 mM, suggesting similar substrate affinity. However, the *k*_cat_ of YhbO (∼1.4 s^−1^) was approximately 3.5-fold higher than that of DJ-1 (∼0.4 s^−1^), resulting in a *k*_cat_/*K*_m_ of ca. 210 M^−1^ s^−1^ for YhbO and ∼55 M^−1^ s^−1^ for DJ-1 (Table 2). These data indicate that YhbO also has higher methylglyoxalase catalytic efficiency than DJ-1. While some kinetic parameters differed slightly from those reported in previous studies (26), our results consistently support the conclusion that YhbO has higher overall catalytic activity than DJ-1 for both PGO and MGO substrates. These findings suggest that YhbO possesses a catalytic profile distinct from the other DJ-1 protein family members, possibly reflecting evolutionary divergence and functional specialization.

**Table 2:** Methylglyoxalase kinetics of DJ-1, YhbO, and the YhbO D78A mutant

Protein	*K*_m_ (M)	*k*_cat_ (s^−1^)	*k*_cat_/*K*_m_ (s^−1^ M^−1^)
WT DJ-1	6.92 ± 0.77 × 10^−3^	0.38 ± 0.022	55.8 ± 3.97
WT YhbO	6.89 ± 1.68 × 10^−3^	1.41 ± 0.193	209.6 ± 41.00
YhbO (D78A)	5.94 ± 0.98 × 10^−3^	0.83 ± 0.096	140.0 ± 9.18

### Molecular modeling of YhbO complexed with its substrate

Although this study (Fig. 1) and a previous study (26) revealed hydratase activity of YhbO for GO and MGO, the molecular mechanisms underlying the α-oxoaldehyde hydrolysis and the specific amino acid residues required for its catalytic activity remain completely uncharacterized. To address this issue, we attempted to model the structure of YhbO complexed with its substrate. The crystal structure of YhbO has already been determined in its substrate-free form and deposited by Claude, J.B., Abergel, C., and Claverie, J.M. (PDB ID: 1OI4). Moreover, structures of a YhbO-like protein from *Arabidopsis thaliana* (AtDJ-1d) complexed with a covalent inhibitor have been reported (30). This covalent inhibitor resembles GO but with one aldehyde group replaced by a carboxylic acid, thereby trapping the molecule in a covalently bound state with a Cys residue. The interactions involving the carbonyl group in this complex are expected to be similar to those between YhbO and GO. Furthermore, residues critical for these interactions are conserved between YhbO and AtDJ-1d.

**Figure 3:**
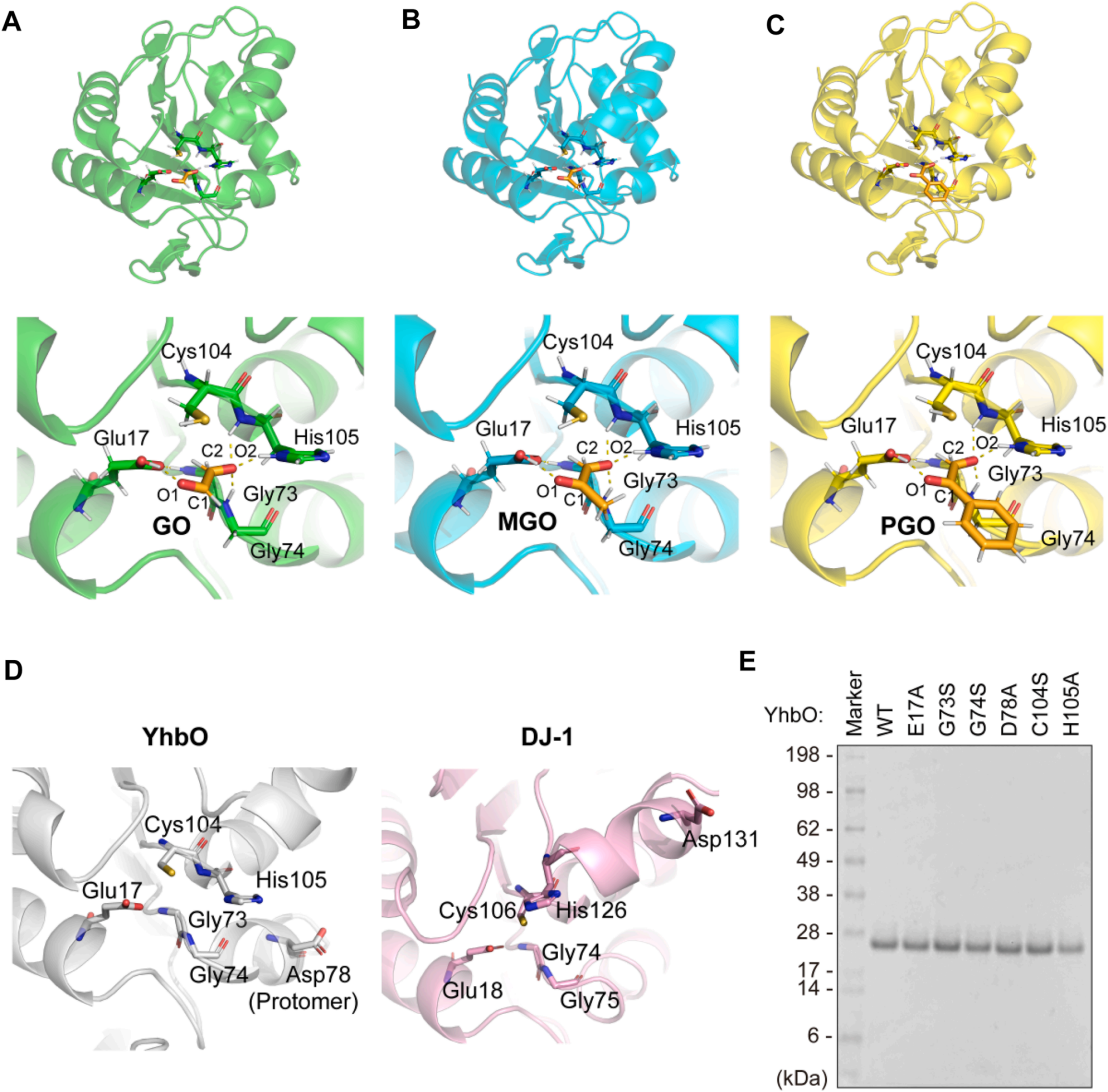
**Structural models of YhbO in complex with α-oxoaldehyde substrates.***A – C*, Molecular models for YhbO recognition of three different substrates. The YhbO–GO, YhbO –MGO, and YhbO –PGO complexes are shown in parallel with the respective substrates shown in *orange*. *D*. Comparison of structures near the catalytic sites of YhbO and DJ-1. E17, G73, and G74, which are located near the catalytic C104 of YhbO, are conserved in DJ-1. In contrast, H105 and D78 (derived from the protomer), which form the putative catalytic triad in YhbO, are not well conserved in DJ-1. *E*. YhbO mutants (15 μg) were separated by SDS-PAGE and stained with CBB.

Based on this information, we constructed model structures of YhbO complexed with GO (Fig. 3*A*), MGO (Fig. 3*B*), and PGO (Fig. 3*C*). In these models, the carbonyl groups of the aldehyde moieties in the substrates form hydrogen bonds with the backbone NH groups between Cys104 and His105, as well as with the backbone NH group of Gly74. Additionally, in the AtDJ-1d structure, the side chain of the residue corresponding to Glu17 in YhbO forms a hydrogen bond with the carbonyl group of the ligand, suggesting that the carboxyl group of Glu17 is protonated. Therefore, our YhbO-ligand complex models were constructed assuming that Glu17 is protonated and forms a hydrogen bond with the substrate.

**Figure 4:**
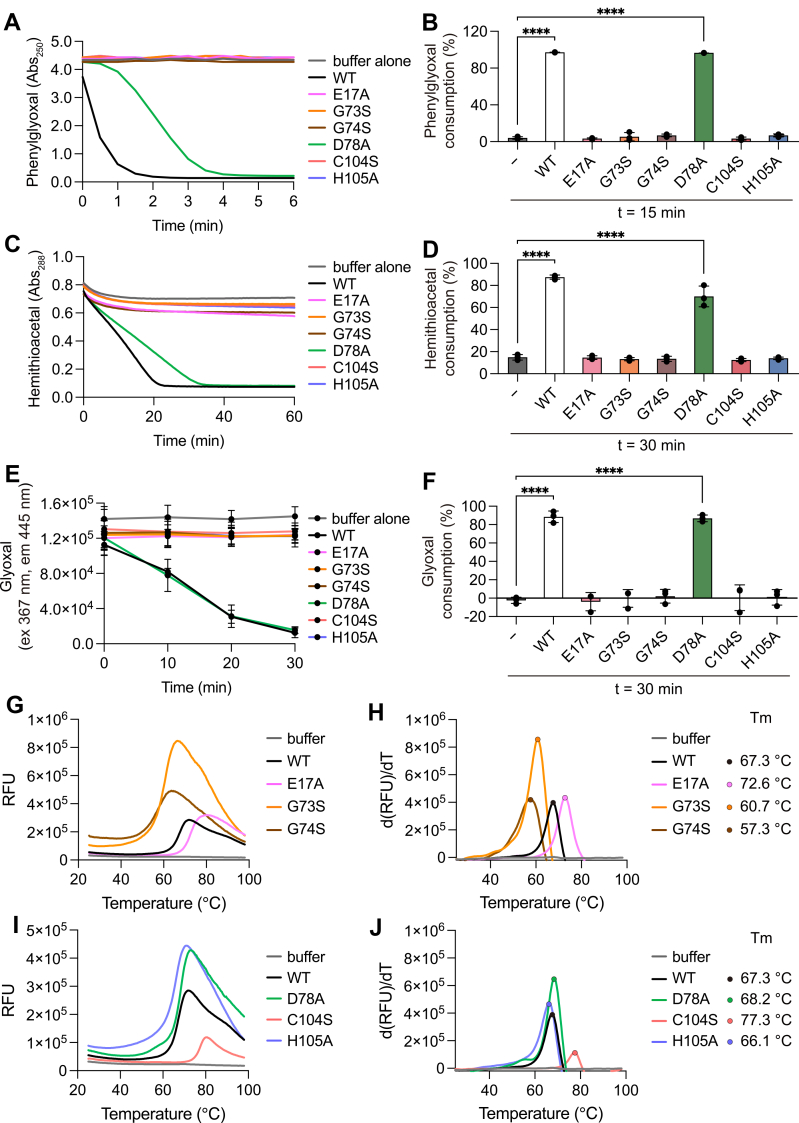
**E17, G73, G74, C104, and H105 are critical for YhbO α-oxoaldehyde hydratase activity.***A*, time course of PGO degradation by WT or mutant YhbO. PGO (1 mM) and YhbO (5 μM) were incubated at 37 °C, and A250 nm was monitored over time. *B*, PGO consumption rates by YhbO mutants were calculated after 15 min. *C*, hemithioacetal (7.5 mM) was monitored in the presence of WT or mutant YhbO (5 μM). *D*, HTA consumption rates were obtained after 30 min. *E*, GO degradation by WT or mutant YhbO. GO (400 μM) with WT or mutant YhbO (0.5 μM) was monitored at the indicated time. *F*, GO consumption rates were determined after 30 min. *G*–*J*. The thermal stability of WT and mutant YhbO was determined. Relative fluorescence units (RFU, *panel G* and *I*) were monitored over a temperature gradient, and the first derivative of RFU with respect to temperature (ΔRFU/ΔT, *panel H* and *J*) was calculated. The Tm of each sample was determined from the peaks of the ΔRFU/ΔT plots. *Graylines* indicate the buffer alone. The data shown in *A*, *C*, and *G*–*J* are representative of three individual experiments. Data in *B*, *D*, *E*, and *F* are the mean ± SD of three replicates (n = 3). ∗*p* < 0.05 using one-way ANOVA with Dunnett’s multiple comparisons test (*B*, *D*, and *F*).

Based on the structural models and the information available on amino acid residues critical for the enzymatic activity of DJ-1 family proteins, we introduced mutations at a number of sites. G73S, G74S, C104S, and H105A mutations were generated because G73–G74, C104, and H105 were predicted to form hydrogen bonds with the substrate. The D78A mutation was prepared because Asp78 from the opposing protomer is predicted to comprise a Cys–His–Asp catalytic triad with Cys104 and His105 (Fig. 3*D*) (25, 31, 32). The E17A mutation was generated because Glu17 and Cys104 are predicted to comprise the unique catalytic center of DJ-1 (20, 28). YhbO proteins harboring these mutations were expressed as His-tagged recombinant proteins and purified. SDS-PAGE analysis confirmed comparable purity and molecular weight (∼20 kDa) across all of the mutants. Because the proteins were recovered as soluble full-length proteins without aggregation, no major folding defects likely occurred (Fig. 3*E*). The α-oxoaldehyde hydrolase activity of the mutants was subsequently assessed (Fig. 4).

### C104, E17, G73, G74, and H105 are essential for the α-oxoaldehyde hydratase activity of YhbO

Phenylglyoxalase activity of the YhbO E17A, G73S, G74S, D78A, C104S, and H105A mutants was first assessed by monitoring the decrease in A250 following incubation with PGO (Fig. 4*A*). WT YhbO rapidly consumed PGO, whereas the E17A, G73S, G74S, C104S, and H105A mutants had minimal or no activity. The D78A mutant exhibited a slight delay in substrate turnover, but its enzymatic activity was comparable to WT (Fig. 4*A*), whereas the E17A, G73S, G74S, C104S, and H105A mutants had less than 5% activity (Fig. 4*B*). Methylglyoxalase activity was monitored at A288 based on HTA adduct formation between MGO and NAC (Fig. 1*D*). The E17A, G73S, G74S, C104S, and H105A mutants failed to reduce absorbance over time, indicating a loss of activity (Fig. 4*C*). The activity of the D78A mutant, in contrast, was almost identical to that of the WT (Fig. 4, *C* and *D*). Glyoxalase activity was examined by measuring the fluorescence decrease (Ex 367 nm/Em 445 nm) of GO–DMB adducts over time (Fig. 1*G*). While WT exhibited robust time-dependent fluorescence reduction, the E17A, G73S, G74S, C104S, and H105A mutants failed to do so, indicating severely impaired activity (Fig. 4, E and *F*). The D78A mutant again retained WT–like glyoxalase activity.

Although these data suggest the importance of E17, G73, G74, C104, and H105 for enzymatic activity, it is possible that the missense mutations disrupted the overall YhbO structure. We thus used a thermal shift assay to monitor the folding state of the mutants (20, 33, 34, 35). In this assay, the protein of interest is incubated with a specialized dye, and its fluorescence is measured in relation to increasing temperature. When the protein unfolds, the exposed hydrophobic surface binds the dye, resulting in increased fluorescence. Unfolding temperatures, which are analogous to melting temperatures (referred to as Tm), indicate the maximum value of the first derivative of the relative fluorescence unit as a function of temperature (dRFU/dT). The thermal shift data confirmed that the E17A, G73S, G74S, C104S, and H105A mutants were folded at room temperature, with the G73S, G74S, D78A, and H105A mutants unfolding around 60 °C (Tm = 60.7 °C for G73S and 57.3 °C for G74S) (Fig. 4, *G* and *H*) or 67 °C (Tm = 68.2 °C for D78A and 66.1 °C for H105A) (Fig. 4, *I* and *J*). For the E17A and C104S mutants, the Tm values exceeded that of WT (Tm = 67.3 °C), suggesting that they were stabilized (Tm = 72.6 °C for E17A and 77.3 °C for C104S) (Fig. 4, *H* and *J*). These results confirmed that the loss of enzymatic activity in the E17A, G73S, G74S, C104S, and H105A mutants was not the result of misfolding but was rather attributable to specific disruption of the catalytic mechanism.

While D78 had been suggested to comprise a Cys–His–Asp catalytic triad with a nucleophilic Cys and neighboring His (25, 31, 32), the D78 residue of YhbO appears to differ from the catalytic triads found in canonical Ser- or Cys-proteases. Even though D78 seemed equivalent to acid residues in catalytic triads, it is not well conserved in DJ-1 or HchA. Moreover, the D78A mutation had little to no effect on hydratase activity with PGO, MGO, or GO (Fig. 4), implying that D78 does not form part of the catalytic triad.

### YhbO H105 is directly involved in the α-oxoaldehyde hydratase catalytic mechanism

**Figure 5:**
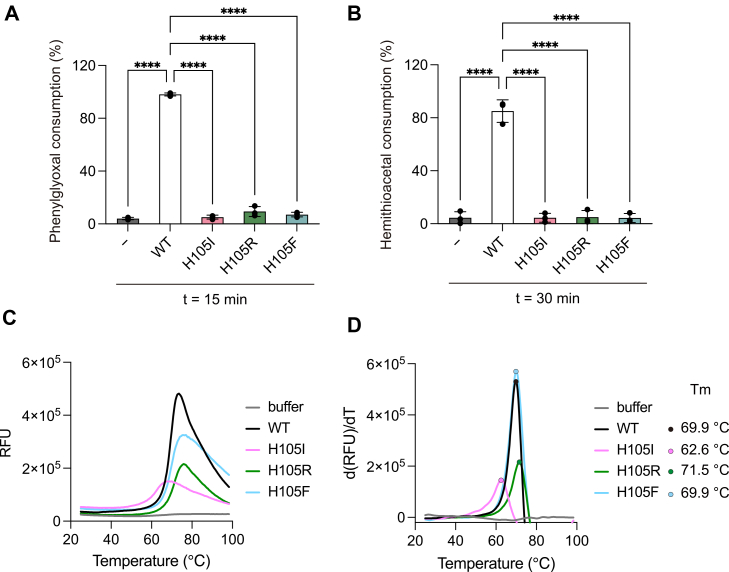
**YhbO H105 is essential for hydrolysis of PGO and MGO.***A*, PGO (1 mM) consumption by WT or H105 mutant YhbO (5 μM) was measured after 15 min. *B,* MGO-derived HTA (7.5 mM) degradation by WT or H105 mutant YhbO (5 μM) was monitored after 30 min. *C, D*, thermal shift assay of YhbO proteins. Relative fluorescence units (RFU, *panel C*) and the first derivative of RFU with respect to temperature (ΔRFU/ΔT, *panel D*) were determined over increasing temperatures. *Graylines* indicate the buffer alone. Tm values correspond to ΔRFU/ΔT maxima. The data shown in *C* and *D* are representative of three individual experiments. Data in *A* and *B* are the mean ± SD of three replicates (n = 3). ∗*p* < 0.05 using one-way ANOVA with Dunnett’s multiple comparisons test (*A* and *B*).

Early structural studies of DJ-1 family proteins postulated that the catalytic triad in both DJ-1 and its PfpI homolog consisted of Cys–His–Glu, with the triad maintaining Cys in its nucleophilic state as the active catalytic center (23). However, later studies suggested that the His residue (H126) near Cys106 in the nucleophile elbow region of DJ-1 did not contribute to the triad (24), and was dispensable for enzymatic activity (6, 30). Similarly, we demonstrated that substituting the His (H186) adjacent to the catalytic Cys (C185) with Ala in HchA did not induce a dramatic loss in either phenylglyoxalase or methylglyoxalase activity (28). Despite these reports, it is clear that YhbO behaves differently as the H105A mutation completely impaired enzymatic activity (Fig. 4, *A*–*F*). Given that a His proximal to the catalytic Cys might be a unique feature of the YhbO enzymatic reaction, we performed additional mutational analyses of H105. Two interpretations could account for this result: (i) the imidazole group of His105 is truly essential because it directly participates in the YhbO hydrolytic reaction; or (ii) steric and hydrophobic effects at this position are critical, such that an equally sized aromatic moiety would suffice to support catalysis. To test the necessity of His, we replaced H105 with Arg, Ile, or Phe and evaluated the activity of each mutant (Fig. 5). Because the imidazole and phenyl rings are comparable in size, retention of activity by H105F would support the steric/hydrophobic hypothesis. Conversely, loss of activity would imply that the basic property of His105 is essential for catalysis. The enzymatic activity of the H105R, H105I, and H105F mutants was examined using PGO and MGO as substrates. Similar to Figure 4, *A*–*D*, A250 for PGO and A288 (HTA with NAC) for MGO were measured. None of the three variants retained phenylglyoxalase or methylglyoxalase activity (Fig. 5, *A* and *B*).

**Figure 6:**
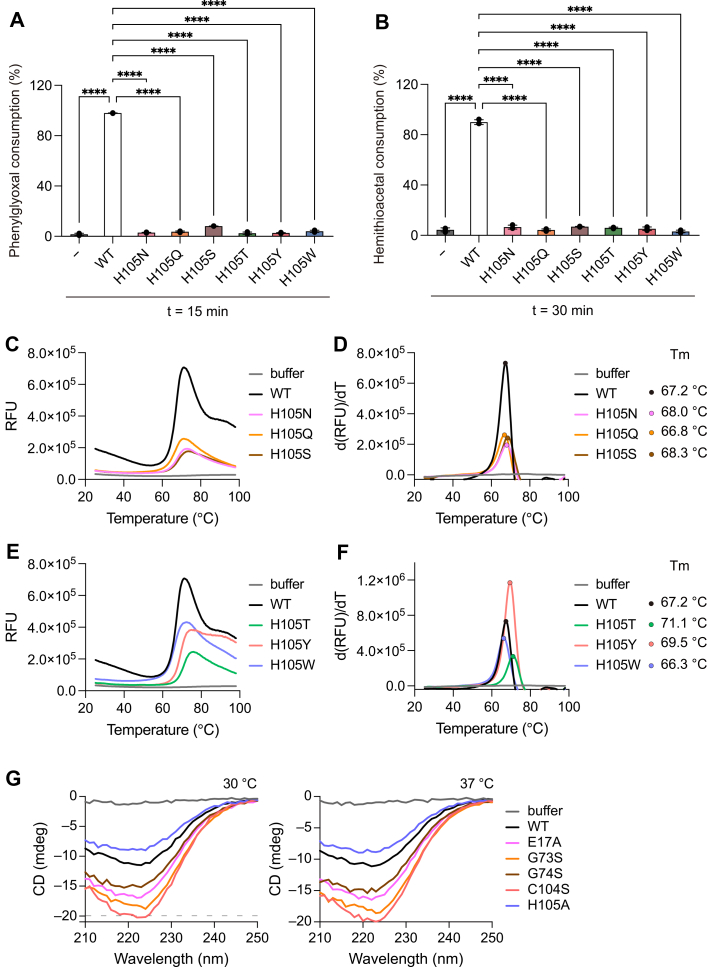
**Histidine-specific requirement of YhbO H105 for α-oxoaldehyde hydratase activity.***A, B,* phenylglyoxalase (*A*) and methylglyoxalase (*B*) activities of YhbO mutants in which His105 was replaced with Asn, Gln, Ser, Thr, Tyr, or Trp. Enzymatic activities were evaluated using the phenylglyoxal and hemithioacetal consumption assays described in Figure 1. Phenylglyoxal consumption was quantified after 15 min and hemithioacetal consumption after 30 min. Data are presented as mean ± SD of three replicates (n = 3). ∗*p* < 0.05 using one-way ANOVA with Dunnett's multiple comparisons test. *C–F*, thermal shift assay of H105 mutants. Relative fluorescence units (RFU) curves for H105N, H105Q, and H105S mutants (*C*) and H105T, H105Y, and H105W mutants (*E*). Corresponding first-derivative plots (ΔRFU/ΔT) used to determine melting temperatures (Tm) are shown in (*D*) and (*F*). *G*, Far-UV CD spectra of WT YhbO and catalytically inactive mutants (E17A, G73S, G74S, C104S, and H105A) at 30 °C and 37 °C. All mutants displayed spectra characteristic of folded proteins, indicating preservation of native-like secondary structure under assay conditions. The data shown in *C-G* are representative of two individual experiments.

To further investigate the chemical requirements at position 105, additional substitutions were introduced. H105 was replaced with Asn (H105N), Gln (H105Q), Ser (H105S), Thr (H105T), Tyr (H105Y), or Trp (H105W), and the enzymatic activities of the resulting mutants were examined using the same phenylglyoxalase and methylglyoxalase assays (Fig. 6). None of these substitutions retained detectable enzymatic activity (Fig. 6, *A* and *B*). Notably, the H105N, H105Q, H105S, and H105T mutants were inactive despite containing side chains capable of forming hydrogen-bonding interactions. Likewise, the H105Y and H105W mutants failed to restore activity despite retaining bulky aromatic side chains. These findings indicate that neither general hydrogen-bonding capability nor aromaticity at position 105 is sufficient to support catalysis. Instead, the results suggest that the unique chemical properties of the histidine imidazole group are specifically required for the α-oxoaldehyde hydratase activity of YhbO. These observations are consistent with a role for H105 in proton transfer during catalysis.

To further investigate whether the loss of activity was due to protein misfolding, we performed thermal shift assays. All mutants, as well as WT YhbO, displayed clear unfolding transitions (Figs. 5*C* and 6, *C*, *E*) and evidence for protein unfolding (Tm) was observed around 70 °C for the WT protein as well as the H105N, H105Q, H105S, H105Y, H105W, and H105F mutants (Figs. 5*D* and 6, *D*, *F*). The H105R and H105T mutants exhibited a slightly increased melting temperature, whereas the H105I mutant exhibited a slightly reduced melting temperature (Figs. 5*D* and 6*F*), suggesting minor stabilization or destabilization, but the overall structural integrity was likely preserved. Collectively, these data show that the enzymatic activities of YhbO were abolished by the H105 mutations, whereas the structural integrity of the mutants was retained. This strongly suggests that H105 itself is indispensable for the YhbO enzymatic reaction, and that the YhbO α-oxoaldehyde hydrolase catalytic mechanism is distinct from that of DJ-1 (see Discussion).

To thoroughly confirm that the loss of enzymatic activity in the E17A, G73S, G74S, C104S, and H105A mutants was not attributable to structural destabilization under assay conditions, we analyzed their secondary structures by far-UV circular dichroism (CD) spectroscopy and their free energies of unfolding (ΔG). All mutants exhibited CD spectra characteristic of folded proteins at both 30 °C and 37 °C, and were broadly similar to that of WT YhbO (Fig. 6*G*), indicating that these proteins retain native-like secondary structures at temperatures used for the enzymatic assays. Interestingly, the E17A, G73S, G74S, and C104S mutants reproducibly showed increased CD signal amplitudes relative to WT, suggesting altered conformational equilibria and/or increased structural stabilization. Consistent with this interpretation, thermal shift assays also suggested that the E17A and C104S mutants are more stable than the WT protein (Fig. 4, *H* and *J*).

**Figure 7:**
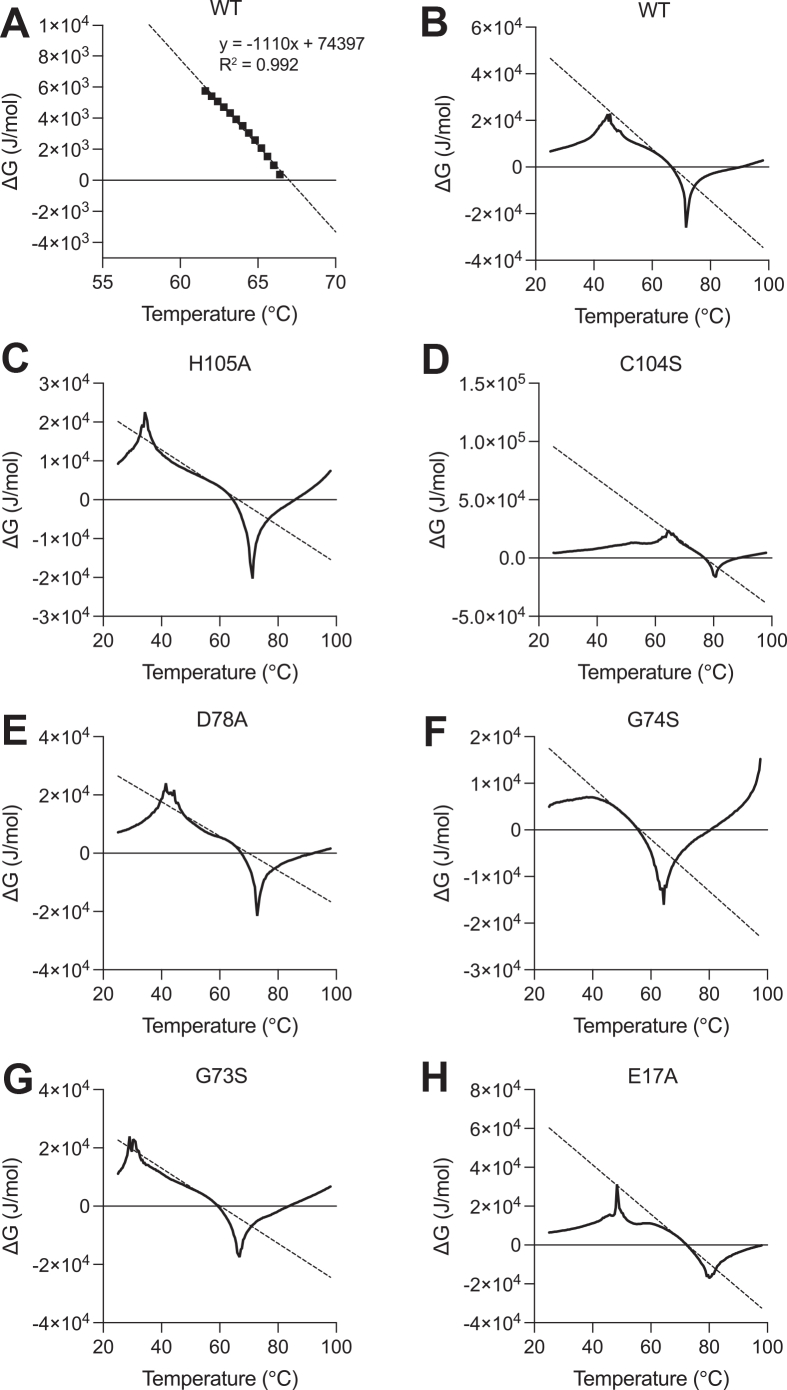
**Estimation of unfolding free energy (ΔG) of YhbO mutants at 37 °C.***A*, representative linear fitting of ΔG values for WT YhbO to determine ΔG at 37 °C. The fraction of unfolded protein (fU) was estimated from thermal shift assay fluorescence data by defining pre-transition and post-transition baselines as fully folded and fully unfolded states, respectively. Equilibrium constants (K) were calculated from fU, and ΔG values were determined using ΔG = −RT lnK. Linear fitting was performed using only data corresponding to 10 to 50% unfolded protein. *B*–*H*. Temperature-dependent ΔG profiles of WT YhbO (*B*), H105A (*C*), C104S (*D*), D78A (*E*), G74S (*F*), G73S (*G*), and E17A (*H*). Solid curves represent calculated ΔG values and dashed lines indicate linear fitting used for extrapolation of ΔG at 37 °C. Experiments to calculate ΔG were repeated independently (n = 2), and the results are summarized in Table 3. Data in *B*–*H* are representative example of two independent experiments.

We next estimated free energies of unfolding (ΔG) for YhbO mutants as reported previously (36, 37). The fraction of unfolded protein (fU) at each temperature was estimated from the thermal shift assay fluorescence data by assuming that the pre-transition and post-transition baselines represent fully folded and fully unfolded protein populations, respectively. Equilibrium constants (K) between folded and unfolded states were determined as K = fU/(1 − fU). The corresponding free energies of unfolding (ΔG) were determined using the relationship ΔG = −RT lnK (Fig. 7). Linear fitting was performed using only the temperature range corresponding to 10 to 50% unfolded protein (Fig. 7*A*) to minimize the impact of protein aggregation and consequent exclusion of the fluorescent dye at high fractions of unfolded protein, and to minimize issues arising from signal-to-noise variations at low proportions of unfolded protein (37). Extrapolation of the resulting thermodynamic parameters to 37 °C revealed that WT YhbO (Fig. 7*B*), H105A (Fig. 7*C*), C104S (Fig. 7*D*), D78A (Fig. 7*E*), G74S (Fig. 7*F*), G73S (Fig. 7*G*), and E17A (Fig. 7*H*) mutants retained positive ΔG values. The estimated ΔG values at 37 °C ranged from 5 to 10 (G74S) to 74 to 79 (C104S) kJ mol^−1^ (Table 3). According to standard thermodynamic considerations, a ΔG value greater than 5.9 kJ mol^−1^ corresponds to a folded-to-unfolded population ratio exceeding 10:1 (38). Thus, almost all proteins examined are predicted to remain overwhelmingly in the folded state at 37 °C. Taken together, CD spectroscopy and thermodynamic analyses also demonstrated that these mutants remain thermodynamically stable under the conditions used for the activity assays.

**Table 3:** ΔG _(folded → unfolded)_ of YhbO mutants (kJ mol^−1^)

Protein	Experiment 1	Experiment 2
WT	31.16	33.32
E17A	45.11	43.05
G73S	14.25	14.99
G74S	5.39	10.82
D78A	19.44	30.07
C104S	73.55	78.95
H105A	14.27	14.32

### Enzymatic characterization of the YhbO D78A mutant

**Figure 8:**
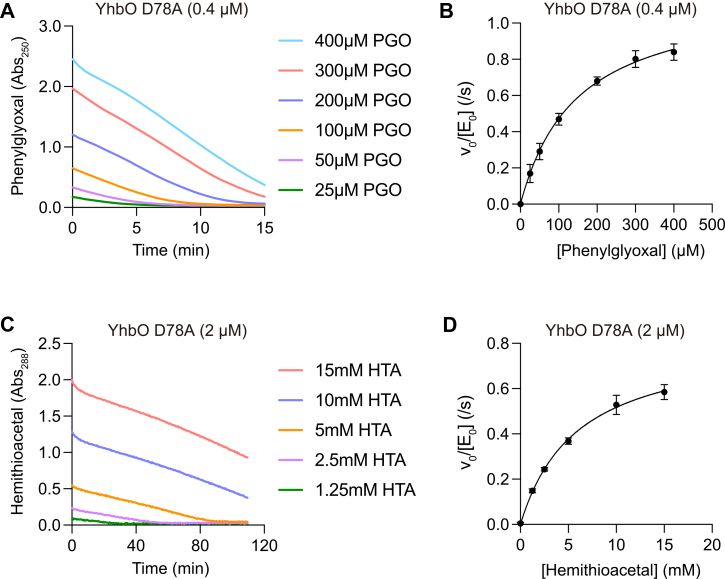
**Kinetic characterization of the YhbO D78A mutant.***A–D*, catalytic activity of YhbO D78A with PGO and MGO. YhbO D78A (0.4 μM) was incubated with the indicated concentrations of PGO or MGO-derived HTA. Absorbance was monitored over time (*A*, *C*), and the corresponding Michaelis–Menten plots were generated (*B*, *D*). The data shown in *A* and *C* are representative of three individual experiments. Data in *B* and *D* are the mean ± SD of three replicates (n = 3). The results are summarized in Tables 1 and 2.

As shown in Figure 4, the YhbO D78A mutant retains detectable α-oxoaldehyde hydratase activity. To further characterize the enzymatic properties of this mutant, we quantitatively analyzed its reaction kinetics as in Figure 2. PGO was incubated with YhbO D78A at varying concentrations and the reduction in A250 over time was measured (Fig. 8*A*). Initial reaction velocities were calculated from the linear range of each kinetic trace and plotted against substrate concentration to generate a Michaelis–Menten curve (Fig. 8*B*). The V_max_ and *K*_m_ kinetic parameters (Table 1) were determined by fitting the data to the Michaelis–Menten equation, and *k*_cat_ values were calculated from V_max_ using the known enzyme concentration [E_0_]. Compared to the WT YhbO, the D78A mutant exhibited a lower *K*_m_ for PGO (∼150 μM vs. ∼370 μM), indicating increased substrate affinity. However, its *k*_cat_ was reduced to about half that of WT (∼2.5 s^−1^ *versus* ∼1.2 s^−1^). As a result, the catalytic efficiency (*k*_cat_/*K*_m_) of the D78A mutant remained comparable to WT YhbO. These results suggest that while the D78A mutation enhances substrate binding affinity, it compromises the catalytic turnover rate, ultimately yielding similar overall activity.

We next evaluated the methylglyoxalase activity of the D78A mutant using MGO- and NAC-derived HTA as a substrate. As with PGO, the initial velocities were calculated for reactions containing various concentrations of HTA (Fig. 8*C*), and the kinetic parameters were obtained *via* Michaelis–Menten analysis (Fig. 8*D* and Table 2). The *K*_m_ for MGO was similar between the D78A mutant (∼6 mM) and WT YhbO (∼7 mM), indicating unchanged substrate affinity. However, the *k*_cat_ of D78A was lower than WT (1.4 s^−1^ vs. 0.8 s^−1^), resulting in a catalytic efficiency that was about 65% of WT. These findings demonstrate that the D78A mutation does not significantly alter substrate binding for MGO but does lead to a modest reduction in catalytic activity. Although D78 was thought to comprise the YhbO catalytic triad (Cys–His–Glu) (Fig. 3*D*), the D78A mutational analyses indicate that this is unlikely.

### Construction of single *yhbO* and double *yhbO/hchA* knockout *E. coli* strains for evaluating α-oxoaldehyde detoxification

**Figure 9:**
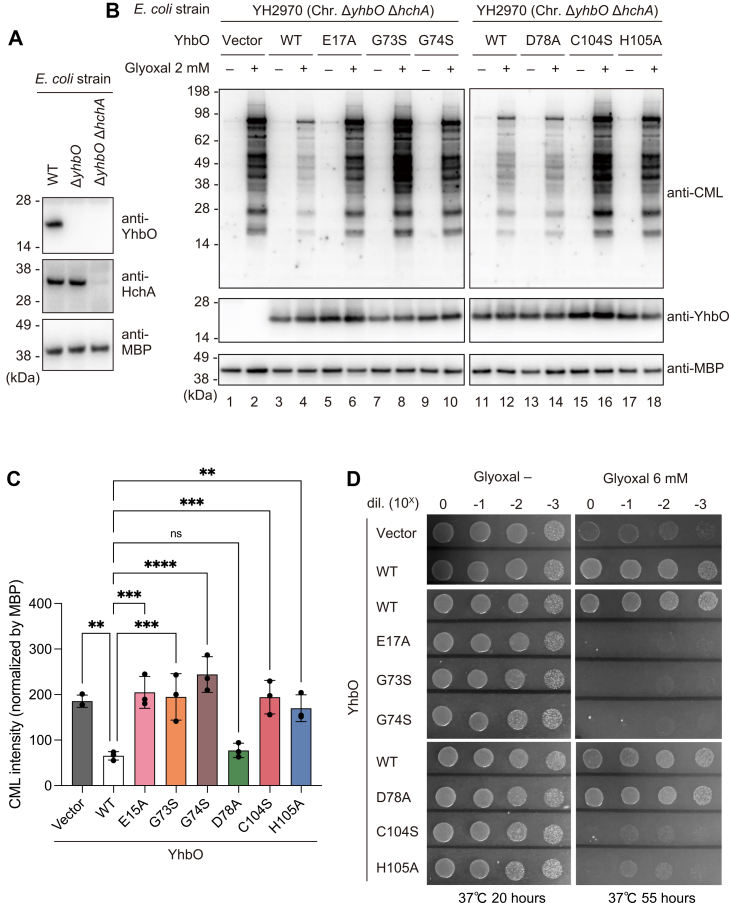
**YhbO protects *E. coli* from glyoxal-induced carbonyl stress.***A*, verification of *yhbO* and *hchA* deletion by immunoblotting. Strains CU141 (WT), YH2949 (Δ*yhbO*), and YH2970 (Δ*yhbO* Δ*hchA*) were grown overnight in M9 minimal medium. The overnight cultures were subsequently diluted 1:20 into fresh M9 medium and incubated for 10 h before harvesting cells for immunoblot analysis. MBP (maltose-binding protein) was used as a loading control. *B, C, and D*, overexpression of YhbO inhibited GO-induced protein modification and conferred cellular resistance to GO. *B*, carboxymethyl-lysine (CML) modification in *E. coli* overexpressing WT or mutant YhbO. WT YhbO, mutant YhbO, or the empty plasmid pTWV228 (vector control) was expressed in *E. coli* strain YH2970 (Δ*yhbO* Δ*hchA*). Cells were cultured in M9-minimal medium supplemented with IPTG and cAMP, and subsequently treated with 2 mM GO. Cell lysates were then collected, and CML-modified proteins were detected by western blotting. *C*, quantification of CML modification in *A*. Data represent the mean ± SD of three individual experiments. *D*, cellular growth in the presence of GO depended on the catalytic activity of YhbO. *E. coli* strains expressing YhbO were cultured as in *A* and then diluted in four steps as indicated in the figure. The diluted cultures were spotted onto M9-minimal medium plates containing 6 mM GO and incubated for 20 to 55 h at 37 °C. The images shown in *B* and *D* are representative images of three individual experiments. *C* is the mean ± SD of three replicates (n = 3). ∗*p* < 0.05 using one-way ANOVA with Dunnett’s multiple comparisons test (*C*).

Numerous DJ-1 family proteins have been reported to exhibit α-oxoaldehyde hydratase activity that detoxifies reactive carbonyl compounds such as GO and MGO *in vitro* (26, 27, 28, 39, 40). However, the physiological relevance of this α-oxoaldehyde hydratase activity remains unclear, as DJ-1-deficient cells do not consistently show increased levels of GO- or MGO-derived modifications (19, 20, 41). Moreover, despite extensive *in vitro* evidence for α-oxoaldehyde hydratase activity in DJ-1 family proteins, little is known about the corresponding *in vivo* and cellular phenotypes. To investigate the physiological role of YhbO-mediated α-oxoaldehyde detoxification, we overexpressed WT or mutant YhbO in *E. coli* and examined their effects on resistance to exogenous GO or MGO. We previously demonstrated that both YhbO and HchA (a paralog of YhbO) retain detoxifying activity against α-oxoaldehydes (28). To eliminate the influence of endogenous YhbO and HchA, we constructed both single (Δ*yhbO*) and double (Δ*yhbO* Δ*hchA*) knockout (KO) strains. Immunoblotting confirmed the loss of endogenous YhbO and HchA expression in these strains (Fig. 9*A*). We then examined how overexpression of YhbO altered resistance to GO and MGO in the medium.

### Mutation of YhbO residues essential for glyoxalase activity reduced resistance to glyoxal stress

GO-induced carbonyl stress results in an accumulation of toxic AGE Nε-carboxymethyl-lysine (CML), which contributes to cell death. To determine if the α-oxoaldehyde detoxification activity of YhbO reduced CML accumulation, we introduced WT and mutant YhbO into the Δ*yhbO* Δ*hchA E. coli* strain and analyzed modification following exposure to GO. Because GO non-specifically generates CML by reacting with Lys residues in proteins, it is also likely to react with amino acids and other amine-containing compounds present in the culture medium. To minimize interference from compounds containing amine in the growth medium, cells were cultured in M9 minimal medium and then exposed to GO. Immunoblotting of cell lysates showed that expression of WT YhbO markedly suppressed CML accumulation (Fig. 9*B*, lanes 4 and 12), as reported previously (26). However, it is unclear if YhbO glyoxalase function contributes to CML suppression. To address this issue, we introduced mutations that impair YhbO glyoxalase activity and assessed effects on CML abundance (see Fig. 4, *E* and *F*). Importantly, among the mutants, D78A retained the suppressive effect (Fig. 9*B*, lane 14), whereas the E17A (lane 6), G73S (lane 8), G74S (lane 10), C104S (lane 16), and H105A (lane 18) mutants failed to reduce CML levels. These findings indicate that the five mutations essential for YhbO glyoxalase activity *in vitro* are also critical for YhbO detoxification of GO *in vivo* (Fig. 9*C*).

We next determined if the YhbO mutants also affected bacterial growth in the presence of GO. The control strain of the Δ*yhbO* Δ*hchA* double knockout *E. coli*, transformed with an empty vector, did not exhibit growth (colony-forming ability) in the presence of 6 mM GO, indicating that this strain was sensitive to excessive GO. In contrast, overexpression of wild-type YhbO restored growth, rendering the *E. coli* strain resistant to 6 mM GO (Fig. 9*D*, top panel). It has already been reported that WT YhbO conferred resistance to GO (26). However, the importance of YhbO glyoxalase activity itself in facilitating bacterial growth had yet to be directly determined. We therefore assessed the impact of the YhbO mutants on cell growth. While the D78A mutant conferred resistance (Fig. 9*D*, bottom panel), the E17A, G73S, G74S, C104S, and H105A mutants largely failed to support growth in response to GO stress (Fig. 9*D*, middle and bottom panels). Collectively, these results demonstrate that GO resistance and suppression of CML modification are dependent on YhbO glyoxalase activity. In conclusion, in the presence of exogenous GO, WT YhbO exerted cytoprotective activity, whereas mutations that impaired glyoxalase activity rendered cells vulnerable to GO-induced damage.

### Mutations in residues essential for methylglyoxalase activity impair resistance to MGO

**Figure 10:**
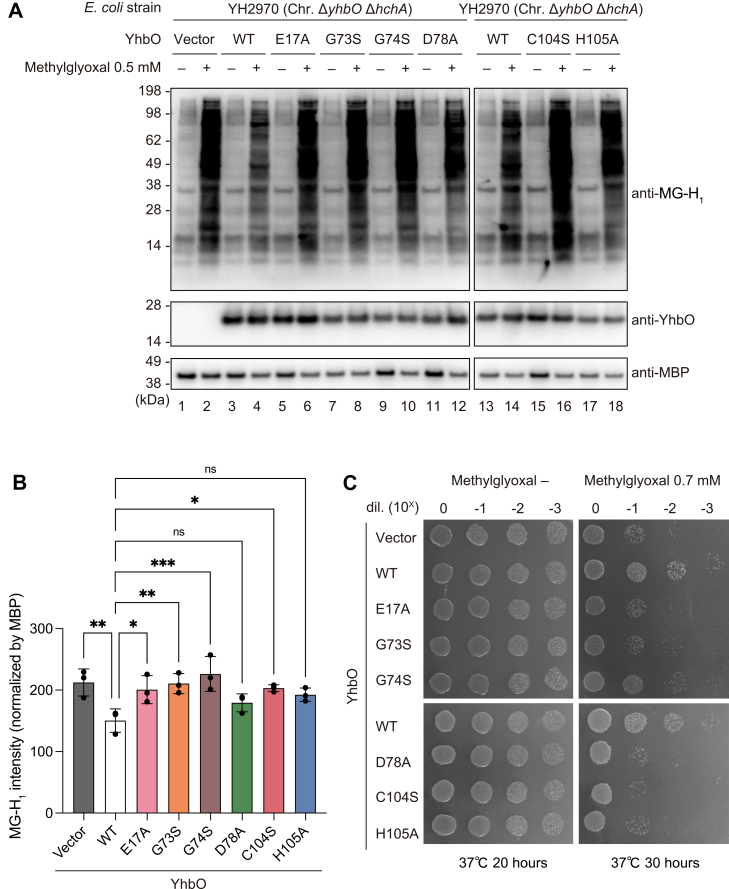
**YhbO enzymatic activity is required to suppress MGO-induced protein modification and cellular growth defect.***A*, *E. coli* expressing WT or mutant YhbO were cultured in media containing 0.5 mM MGO. Proteins modified by MGO were detected using an anti-MG-H1 antibody. *B*, The levels of MGO-modified proteins in *A* were quantified. Data represent the mean ± SD of three individual experiments. *C*, *E. coli* survival in M9-minimal medium with 0.7 mM MGO was assessed. The images shown in *A* and *C* are representative images of three individual experiments. *B* is the mean ± SD of three replicates (n = 3). ∗*p* < 0.05 using one-way ANOVA with Dunnett’s multiple comparisons test (*B*).

MGO exposure also causes carbonyl stress and leads to an accumulation of toxic MGO-derived AGEs, such as methylglyoxal-hydroimidazolone (MG-H1) and carboxyethyl-lysine (CEL). Overexpression of YhbO has been shown to confer resistance to MGO-induced growth inhibition (26). However, as with the previous section, it is unclear if mutations that impair YhbO enzymatic activity (*i.e.*, methylglyoxalase activity) compromise the protective effect. To determine if MGO resistance is dependent on enzymatic activity, we expressed WT or mutant YhbO proteins in *E. coli* and analyzed MG-H1 levels after MGO treatment. Expression of WT YhbO slightly suppressed MG-H1 accumulation (Fig. 10*A*, compare lanes 2–4), whereas the E17A (lane 6), G73S (lane 8), G74S (lane 10), and C104S (lane 16) mutants failed to do so. Although the band intensities for the D78A and H105A mutants were intermediate relative to bands for WT and the other mutants (Fig. 10*A*, lanes 12 and 18), statistical analysis of repeated experiments failed to reveal a significant difference between the D78A and the H105A mutants and WT YhbO (Fig. 10*B*).

We also performed bacterial growth assays using MGO-containing agar plates to further examine the effects of the YhbO mutants on MGO resistance. As with the previous section, the control strain of the Δ*yhbO* Δ*hchA* double knockout strain harboring an empty vector exhibited impaired growth in the presence of 0.7 mM MGO, indicating that this strain was sensitive to excessive MGO. In contrast, overexpression of wild-type YhbO made the strain resistant to 0.7 mM MGO and restored colony-forming ability (Fig. 10*C* upper panel), as has been reported (26). To examine the importance of YhbO methylglyoxalase activity in facilitating bacterial growth under excessive MGO, we performed mutational analyses of YhbO. The protective capacity of the G74S mutant was reduced, and the E17A, G73S, D78A, C104S, and H105A mutants provided no protection (Fig. 10*C*). These findings indicate that the catalytic Cys and surrounding residues are essential for both suppression of MG-H1 accumulation and protection from MGO-induced cytotoxicity. Together, the data support a functional link between YhbO methylglyoxalase activity and its physiological role in mitigating exogenous MGO stress.

### Physiological relevance of YhbO–associated glyoxalase activity is limited under steady-state conditions

**Figure 11:**
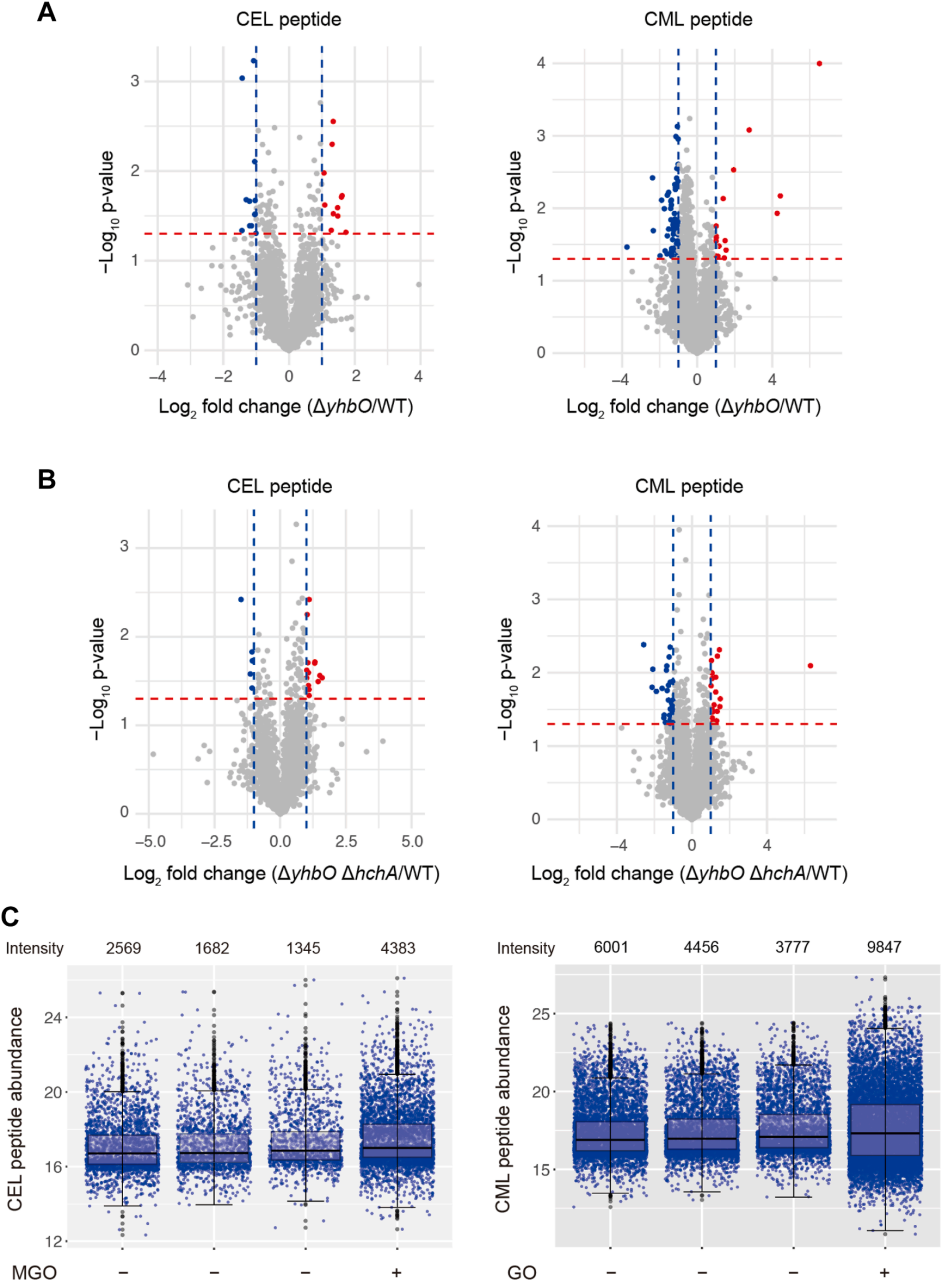
**α-oxoaldehyde-modified peptides do not accumulate following deletion of YhbO.***A*, proteins were digested with trypsin, and carboxyethyl-lysine (CEL) modified peptides (*left*) or carboxymethyl-lysine (CML) modified peptides (*right*) were enriched and analyzed by LC-MS/MS. Volcano plot indicates the log2 fold changes in CEL or CML peptide (*x*-axis) and the -log10 of the *p* values (Student’s *t* test) (*y*-axis). CEL- or CML-modified peptides significantly altered in YhbO-deleted strains compared to WT strains (log2FC > 1 or < −1, *p* < 0.05) are shown as *red* (increase) or *blue* (decrease) *circles*. Mean fold changes and *p*-values were calculated from three biological replicates (n = 3). *B*, Volcano plot of CML and CEL peptides prepared from YhbO and HchA double deletion strains relative to WT strains as in *A*. *C*. Abundance of CEL (*left*) and CML (*right*) peptides in the double deletion strains cultured in medium containing 0.2 mM MGO or 1 mM GO. Box plots show the distribution of values. *Blue* dots indicate individual data points, while *greydots* represent outliers.

We next examined the contribution of YhbO to modifications derived from endogenous GO (*i.e.*, CML) and MGO (*i.e.*, CEL) under normal conditions. To enrich for peptides carrying CML or CEL, *E. coli* cell lysates from WT and *yhbO* KO cells were digested with trypsin and processed with a PTMScan CML/CEL kit. The enriched samples were then analyzed by mass spectrometry and compared between WT and *yhbO* KO cells. As shown in Figure 11*A*, peptides with either the CML or CEL modification were not elevated in the *yhbO* KO cells. Previously, we confirmed that HchA also has glyoxalase activity (28), and thus the absence of a phenotype in the *yhbO* KO cells may be due to functional redundancy between HchA and YhbO. To rule out this possibility, we made a double KO strain lacking both *yhbO* and *hchA* (Fig. 9*A*) and repeated the experiment. Dysfunction of both *yhbO* and *hchA* had no effect, as there was no increase in peptides with CML or CEL modifications (Fig. 11*B*). To confirm that elevated MGO or GO levels can increase CML and CEL modifications, the double KO cells were treated with exogenous GO or MGO, and the cell extracts were analyzed as before. This time, we observed an increase in both modification types following MGO and GO treatment (Fig. 11, *C* and *D*), as shown by the immunoblotting in Figures 9*B* and 10*A* (compare lanes 1 and 2). We think the incongruency between the two experiments (Figs. 9 and 10 *versus* Fig. 11) likely reflects redundancy in the cellular MGO and GO detoxification mechanisms, with the Glo1/2 system (1, 42, 43) serving as the most critical α-oxoaldehyde hydratase in cells (see Discussion).

**Figure 12:**
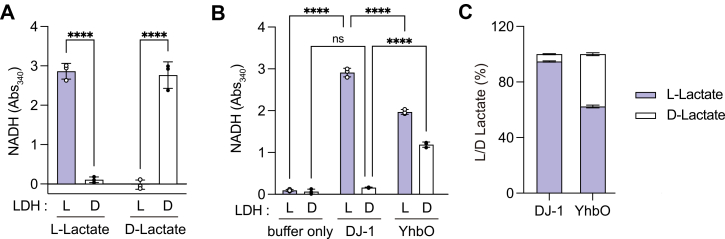
**YhbO produces both L-lactate and D-lactate from MGO.***A*, two enantiomers of lactate (L-lactate and D-lactate) could be distinguished by enantiomer-specific D-LDH and L-LDH. *B,* MGO (7.5 mM) was incubated with WT DJ-1 or YhbO (20 μM) for 1 h at 37 °C. L-LDH or D-LDH along with 1.4 mg/ml NAD+ was added, and the reaction mixture was incubated for 30 min. NADH formation was monitored by A340. *C*, The L-lactate to D-lactate formation ratio by DJ-1 or YhbO was calculated from the data in *A*. All data represent the mean ± SD of three individual experiments (n = 3). ∗*p* < 0.05 using two-way ANOVA with Bonferroni's multiple comparisons test.

Since DJ-1 converts MGO to L-lactate (27), we expected that YhbO might also convert MGO to lactate. Although we showed YhbO-mediated consumption of MGO (Figure 1, Figure 2, Figure 3, Figure 4, Figure 5, Figure 6), those assays did not examine lactate production. To examine this conversion and determine if YhbO generates L-lactate or D-lactate, we performed a classic lactate-monitoring assay. In this assay, lactate produced by DJ-1 or YhbO is converted to pyruvate by lactate dehydrogenase (LDH), which is coupled to the generation of a reduced nicotinamide adenine dinucleotide (NADH) from oxidized nicotinamide adenine dinucleotide (NAD). Accordingly, the presence of lactate can be detected as the characteristic absorbance of NADH at 340 nm. By using enantiomer-specific LDHs (D-LDH and L-LDH), the two enantiomers of lactate could be distinguished (Fig. 12*A*). Next, MGO was incubated with WT DJ-1 or YhbO for 1 h at 37 °C, and then L-LDH or D-LDH along with NAD+ was added, and the reaction mixture was incubated for 30 min. Subsequently, NADH formation was monitored by A340. When DJ-1 was incubated with MGO, the production of only L-lactate was observed (Fig. 12*B*). In contrast, under the same experimental conditions, YhbO produced both L-lactate and D-lactate (Fig. 12*B*). Thus, while DJ-1 converts MGO to L-lactate, YhbO converts MGO to both L- and D-lactate (Fig. 12*C*). These results further demonstrate that the catalytic mechanisms underlying DJ-1 and YhbO differ.

**Figure 13:**
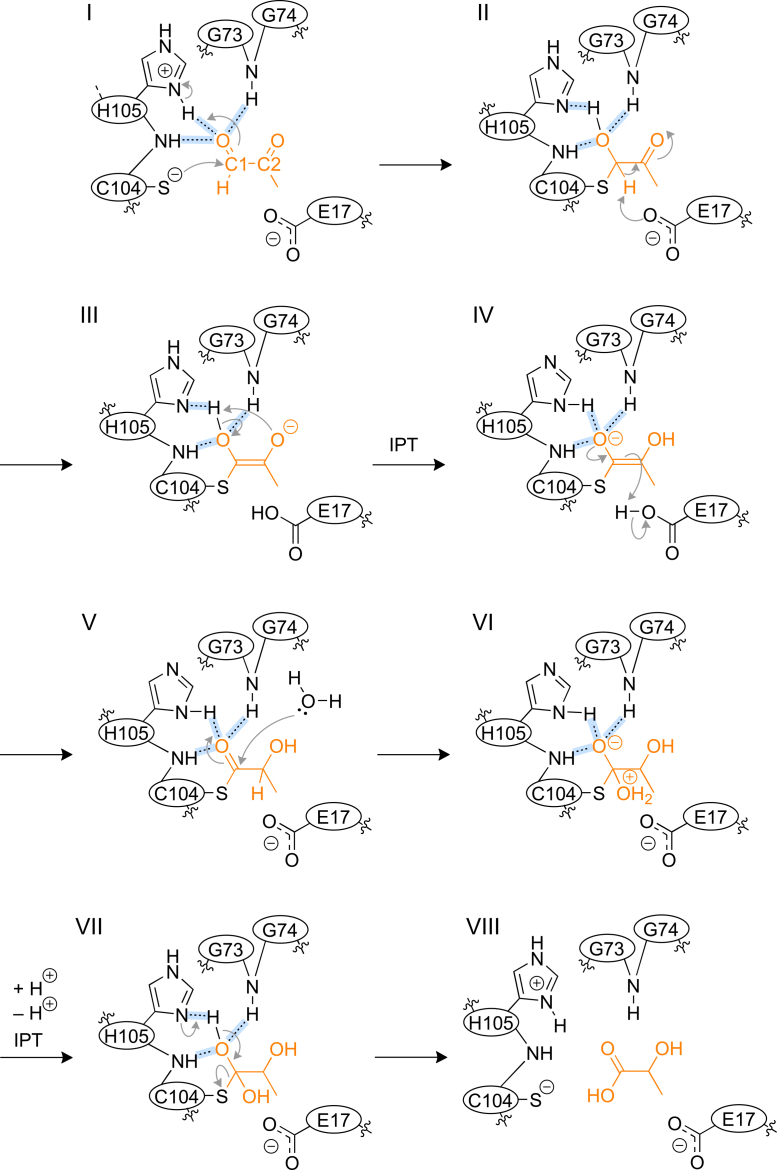
**Proposed reaction mechanism for YhbO–mediated MGO hydrolysis.** IPT denotes the intramolecular proton transfer of the imidazole NH proton. Refer to the main text for details of each reaction step.

Finally, building on insights gained from the mechanism proposed for AtDJ-1d-mediated MGO degradation and from our mutational analysis, we considered the catalytic steps driving YhbO α-oxoaldehyde hydrolase function. We surmise that the detoxification of MGO by YhbO proceeds through three steps: (i) formation of an HTA intermediate (Fig. 13, I–II), (ii) isomerization leading to thioester formation (Fig. 13, II–V), and (iii) hydrolysis of the thioester to produce lactate (Fig. 13, V–VIII). The reaction [especially step (ii)] may be governed by the unique ability of imidazole to act as both a hydrogen-bond donor and acceptor; the imidazole NH undergoes intramolecular proton transfer (IPT) to stabilize the enol or enolate form at the C1 oxygen.

In the first step, the Cys104 thiolate, acting as a nucleophile, attacks the carbonyl carbon (C1) of MGO, while the imidazolium group of His105 donates a proton, resulting in the formation of an HTA intermediate (Fig. 13, I–II). The transient hydroxyl group (OHC1) in the HTA is expected to be stabilized through hydrogen bonds with the backbone amide NH of Gly74 and His105, and the imidazole ring of His105. In the second step, an isomerization reaction takes place. Glu17 abstracts the proton bound to C1 of the HTA (Fig. 13, II), thereby generating a *cis*-enediolate intermediate (Fig. 13, III). Subsequently, the proton from the C1-enol group migrates to the enolate oxygen at C2, resulting in the conversion of C1 into an enolate and C2 into an enol (*i.e.*, an intramolecular proton transfer). The negative charge on the C1-enolate is likely stabilized by hydrogen bonds with nearby residues such as the amide NH of Gly74 and His105. Tautomerization then converts the C1-enolate into a carbonyl group, and the C2 atom becomes protonated by the Glu17 carboxyl group. Consequently, an acyl-enzyme intermediate is formed, in which the substrate is covalently linked to the enzyme *via* a thioester bond (*i.e.*, an S-lactyl-cysteine intermediate; Fig. 13, V). In the third step, the thioester bond undergoes hydrolysis. Nucleophilic attack by a water molecule on the carbonyl carbon of the thioester within the acyl-enzyme intermediate results in formation and subsequent collapse of a tetrahedral intermediate. Through subsequent proton transfers (Fig. 13, V–VII), the enzyme is regenerated, and MGO is converted to lactate.

## Discussion

In this study, we investigated the enzymatic and physiological properties of YhbO, an *E. coli* homolog of DJ-1. Our analyses revealed three important aspects of YhbO α-oxoaldehyde hydratase activity: (1) the strict requirement of H105 for catalysis, (2) enzymatic activity is indispensable for protecting cells from reactive carbonyl species, and (3) structural modeling supports a credible catalytic mechanism. These aspects are discussed in detail below.

### Unique his requirement for YhbO catalyzed α-oxoaldehyde hydrolysis

We introduced a variety of mutations into YhbO and evaluated their α-oxoaldehyde hydratase activity using newly established real-time spectrophotometric and fluorometric assays. We also monitored the growth of *E. coli* under GO and MGO stress to determine if the mutants characterized *in vitro* also contributed to cellular protection. From these experiments, we identified that a unique feature of YhbO is the critical importance of His105. We unexpectedly found that the H105A mutation abolished the enzymatic activity of YhbO both *in vitro* and *in cells* (Figs. 4, 9 and 10) and that more conservative substitutions likewise eliminated enzymatic activity (Figs. 5 and 6). Notably, the impact of the H105F/Y/W mutations highlighted the catalytic importance of the imidazole ring itself as opposed to general hydrophobic and/or steric effects. These results provide important insights into the YhbO catalytic mechanism.

The His dependence of YhbO distinguishes it from other DJ-1 family proteins. DJ-1 was classified in the highly conserved PfpI/Hsp31/DJ-1 superfamily, which is characterized by a catalytic triad motif consisting of Cys-His-Asp/Glu. Consequently, research has largely focused on the His residue proximal to the predicted catalytic Cys. However, in DJ-1, the His (H126) near the catalytic Cys (C106) is not essential for enzymatic activity (6, 30). Instead, the catalytic mechanism is primarily explained by the catalytic Cys and a nearby Glu (E18) (44). Similarly, in HchA, substituting the equivalent His (H186A) had little impact on activity (28). In contrast, multiple substitutions of YhbO H105 abolished enzymatic activity, demonstrating a unique catalytic requirement among these enzymes. Furthermore, the enantiomer ratio of lactate, the final product after MGO detoxification, differs between YhbO and DJ-1 (Fig. 12), further supporting the hypothesis that their catalytic mechanisms are distinct.

The enzymatic activity of YhbO is directly correlated with its physiological role. WT YhbO enhanced cellular resistance to GO and MGO stress and suppressed the accumulation of AGEs, including CML and MG-H1 (Figs. 9 and 10). In contrast, YhbO mutants with reduced hydratase activity failed to provide protection. Although YhbO overexpression was reported to suppress the accumulation of CML and MG-H1 (26), the study did not clarify whether the phenotype was specifically attributable to the enzymatic activity of YhbO. Our results clearly establish that the physiological effects of YhbO, protection against cytotoxic reactive carbonyl species, are dependent on its enzymatic activity. While we clearly observed that overexpression of YhbO conferred cellular resistance to GO and MGO by degrading the reactive carbonyl species (Figs. 9 and 10), the abundance of GO-derived (CML) or MGO-derived (CEL) modifications in the *yhbO* KO and *yhbO/hchA* double KO strains was comparable to controls (Fig. 11), indicating that endogenous YhbO is dispensable for the degradation of GO and MGO generated within cells under steady-state conditions. We surmise that the canonical GO/MGO detoxification system mediated by Glo1 and Glo2 (3, 43) is more sufficient for overcoming the accumulation of GO and MGO under steady-state conditions. Indeed, even though we revealed the clear methylglyoxalase activity of YhbO (Table 2), its *k*_cat_ (approx. 1.5 s^−1^) is ∼700 times lower than that of Glo1 (approx. 1 × 10^3^ s^−1^) and YhbO’s *k*_cat_/*K*_m_ (s^−1^ M^−1^) is ∼5 × 10^4^ lower than that of Glo1 (45). YhbO does not serve as the primary pathway for α-oxoaldehyde detoxification under basal conditions; however, it becomes particularly relevant under conditions of elevated carbonyl stress. YhbO thus may act as a backup enzyme that supplements the canonical glyoxalase I/II pathway when its capacity is exceeded.

### Molecular mechanism underlying YhbO catalysis of α-oxoaldehyde hydrolysis

Given that the molecular mechanisms for various types of glyoxalases (*i.e.*, glyoxalase I–III) have been well studied (1, 42, 43), they can provide a useful framework for elucidating the MGO detoxification process mediated by YhbO. In the classical pathway catalyzed by mammalian Glo1/2, GloA/B in *E. coli*, or GlxA/B in *Bacillus subtilis*, the thiol cofactors, such as glutathione (for Glo1/2 and GloA/B) and bacillithiol (for GlxA/B), act as nucleophiles to initiate the reaction (1, 46). In contrast, DJ-1 family proteins, including YhbO, do not use these cofactors to catalyze MGO detoxification. Rather, their catalytic mechanism is more analogous to that of Cys hydrolases. Namely, the catalytic Cys directly attacks the carbonyl carbon of MGO, forming a hemithioacetal (HTA) and enediol intermediate that rearranges into a thioester. Hydrolysis of this thioester produces lactate, while transition states are stabilized by an oxyanion hole formed by a conserved pair of Gly. Building on this framework, we propose that YhbO detoxifies MGO using a well-conserved Glu (E17), the Gly pair (G73–G74), a catalytic Cys (C104), and a neighboring His (H105). Importantly, our model is reinforced by *in silico* simulations using the AtDJ-1d–glyoxylate complex as a structural template (30). The glyoxylate is structurally similar to GO, albeit with one aldehyde group replaced by a carboxylic acid. Therefore, although it can mimic genuine substrates such as GO and MGO, it halts the AtDJ-1d reaction by covalently binding to the catalytic Cys. Based on the structural insights obtained from our structural modeling (Fig. 3), we have proposed a rational catalytic mechanism for YhbO (Fig. 13).

YhbO is predicted to convert MGO into lactate through three sequential steps: (i) formation of an HTA intermediate, (ii) isomerization leading to thioester formation, and (iii) hydrolysis of the thioester.

In the first step, C104 attacks the carbonyl carbon of MGO and receives a proton from the H105 imidazolium group, resulting in the formation of an HTA intermediate (Fig. 13, I–II). The hydroxyl group of this HTA is likely stabilized by hydrogen bonds with the backbone amide NH of G74 and the H105 imidazole group. The second step consists of an isomerization reaction. After E17 facilitates the formation of a *cis*-enediolate intermediate, an intramolecular proton transfer converts C1 into an enolate and C2 into an enol. The negative charge on the C1 enolate is predicted to be stabilized by hydrogen bonds from the amide NH and the H105 imidazole group. Subsequently, the C1 enolate is converted to a carbonyl group, and C2 is protonated by E17, leading to the formation of a thioester intermediate (acyl-enzyme intermediate). In the final step, a water molecule attacks the carbonyl carbon of the thioester, which sequentially forms and collapses a tetrahedral intermediate, and lactate is generated from YhbO catalysis of MGO. This molecular model is consistent with our mutational analyses as the loss of enzyme activity was observed for the E17A, G73S, G74S, C104S, and H105A mutants (Figs. 4, 9 and 10). CD spectroscopy and thermodynamic analyses demonstrated that these mutants remain folded and thermodynamically stable at 37 °C (Figs. 6 and 7), suggesting that these mutations directly disrupt catalytic function rather than indirectly affecting protein stability. Most importantly, our model provides a mechanistic role for H105 in YhbO, which is not critical for catalysis in either DJ-1 or HchA.

By integrating structural modeling with mutational analyses performed both *in vitro* and *in cell*, we established a likely reaction mechanism for YhbO. Even though DJ-1, HchA, and YhbO possess α-oxoaldehyde hydratase activity, the proposed YhbO catalytic mechanism is distinct from that used by DJ-1 and HchA. In conclusion, the results of this study provide a comprehensive understanding for how YhbO detoxifies reactive carbonyl species.

## Experimental procedures

### Plasmids

Plasmids for N-terminal His-tagged DJ-1 and C-terminal His-tagged YhbO expression were constructed by cloning the relevant genes into pET28a or pET21a vectors as previously reported (20, 28, 47). To assess *E. coli* growth and modification, the *yhbO* gene was cloned into the pTWV228 vector (48, 49). Plasmids expressing various YhbO mutants were generated using a standard 2-step PCR method. All mutations were confirmed by Sanger DNA sequencing.

### Purification of recombinant proteins

Expression plasmids for His-tagged DJ-1 and YhbO were transformed into *E. coli* BL21 (DE3) + RIL (Agilent Technologies). A single colony was inoculated into 2 ml of LB medium containing 25 μg/ml of kanamycin or 100 μg/ml of ampicillin and cultured overnight at 37 °C and 170 rpm. The preculture was added to 100 ml LB and cultured for 2 h at 32 °C and 120 rpm. Once the culture had reached an A600 of 0.3 to 0.5, 0.3 mM IPTG was added, and the culture was incubated for another 3 h under the same conditions. Bacteria were pelleted by centrifugation at 5800 × *g* for 10 min with the resulting pellets resuspended in lysis buffer [20 mM Tris buffer (pH 7.5), 200 mM NaCl, 10 mM β-mercaptoethanol, 1 μg/ml lysozyme (Wako), 1 μg/ml DNase I, and 5 mM MgCl2] and sheared using a sonicator. Cellular debris was removed by centrifugation at 5800 × *g* for 10 min at 4 °C, and the resulting supernatant was recovered. His-tagged recombinant proteins were purified using standard procedures with nickel-agarose (Ni-NTA Agarose, Qiagen) and associated elution buffers [20 mM Tris buffer (pH 7.5), 200 mM NaCl, 10 mM β-mercaptoethanol, and 250 to 500 mM imidazole]. To remove imidazole, the eluted samples were dialyzed twice against 1 l of dialysis buffer [Tris buffer (pH 7.5), 20 mM NaCl, and 1 mM DTT] for at least 4 h using 10 kDa molecular weight cut-off cassettes (Thermo Fisher Scientific). Protein purity was confirmed by Coomassie Brilliant Blue–stained SDS-PAGE gels. Protein concentrations were determined using a BCA protein assay kit (Pierce).

### Measurement of α-oxoaldehyde hydratase activity

To measure α-oxoaldehyde hydratase activity, PGO, MGO, and GO were used. MGO and GO are physiological substrates, whereas PGO serves as a mimetic analogue. Because PGO has an absorbance maxima at 250 nm, its consumption can easily be monitored in real time, making it an ideal model substrate for assessing α-oxoaldehyde hydratase activity. To assess phenylglyoxalase activity, 5 μM purified protein (WT DJ-1, WT YhbO, or the following YhbO mutants - E17A, G73S, G74S, D78A, C104S, H105A, H105I, H105R, or H105F was incubated with 1 mM PGO (TCI Chemicals) in 20 mM sodium phosphate buffer (pH 7.0) at 37 °C. The consumption of PGO was monitored by a reduction in A250 over time (28). For assessing methylglyoxalase activity, the HTA was generated by pre-incubating 7.5 mM MGO (Sigma-Aldrich) and 7.5 mM N-acetyl-L-cysteine (Sigma-Aldrich) at room temperature for 30 min in 50 mM sodium phosphate buffer (pH 7.0). Recombinant proteins (5 μM) were then added to the reaction mixture as previously reported (27, 28), and HTA levels were monitored over time *via* A288. To assess enzymatic activity with GO as a substrate, the GO concentration was quantified using the fluorescent derivative 1,2-diamino-4,5-methylenedioxybenzene (DMB), as previously reported (28). GO (400 μM) and recombinant protein (1 μM) were reacted at 37 °C for 0, 10, 20, or 30 min in 20 mM sodium phosphate buffer (pH 7.0). Fresh DMB solution [7 mM DMB (Dojindo), 28 mM sodium dithionite (Sigma), and 1 M β-mercaptoethanol] was then added to the reacted mixture and incubated at 60 °C for 40 min. After cooling the mixture on ice, the GO–DMB adduct was quantified by measuring excitation at 367 nm and emission at 445 nm. All assays were conducted using a spectrophotometer (NanoDrop) or an Enspire plate reader.

### Determination of enzyme kinetics

Each purified protein was mixed with 5 to 8 different concentrations of PGO or HTA, and changes in A250 or A288 were determined over time. The initial velocities were obtained *via* linear regression of the acquired data. To calculate V_max_ and *K*_m_, the initial velocity data were fitted to a Michaelis–Menten plot using Prism 10 (GraphPad Software). The catalytic constant *k*_cat_ is equal to V_max_ divided by the purified protein concentration. All measurements were repeated at least three times, and either the representative data or average values were reported.

### L/D lactate assay

The chirality of lactate produced from MGO by YhbO was assessed as previously reported (27, 30). For lactate production, 20 μM DJ-1 or YhbO and 7.5 mM MGO were incubated in 50 mM sodium phosphate buffer (pH 7.0) at 37 °C for 1 h. Reaction mixtures (20 μl) were sampled and mixed with 250 μl of glycine-hydrazine buffer (pH 9.2) (G5418, Sigma-Aldrich), 25 μl of 17 mg/ml β-NAD + solution (Oriental Yeast), and 2 μl of recombinant L-LDH (10,000 U/ml, Oriental Yeast) or D-LDH (10,000 U/ml, Oriental Yeast) at room temperature for 30 min. To validate the specificity of the assay, we confirmed that L-LDH and D-LDH selectively react with L-lactate and D-lactate, respectively. This was achieved by performing control experiments in which 7.5 mM L-lactic acid (Wako) or D-lactic acid (Tokyo Chemical Industry) was added directly to the reaction mixture in place of the enzymatically produced products. Lactate production was quantified based on NADH production and A340 monitored with a spectrophotometer (Nano-drop).

### Protein thermal shift assay

To monitor the thermal stability of recombinant proteins, WT and mutant YhbO proteins (30 μM) were incubated with Thermal Shift™ Buffer and Protein Thermal Shift™ Dye (ThermoFisher Scientific). Fluorescence intensity was measured using the ROX reporter from a StepOnePlus™ Real-Time PCR system (Applied Biosystems) and a ramp rate of 0.05 °C/s per step (25–99 °C). The melt temperatures were determined using Protein Thermal Shift™ Software (ThermoFisher Scientific).

### Construction of strains

*E. coli* gene deletion strains YH2949 (Δ*yhbO*) and YH2951 (Δ*hchA*) were constructed as follows. The Δ*yhbO*::*kan* region from JW5529 or the Δ*hchA*::*kan* region from JW1950 (50) was introduced into CU141 by P1 transduction, respectively, and the *kan* cassette of each resulting strain was deleted using pCP20, as described previously (51). The double deletion strain YH2970 (Δ*yhbO* Δ*hchA*) was constructed by repeating P1 transduction of the Δ*hchA*::*kan* marker from JW1950 into YH2949 with subsequent *kan* cassette deletion. Successful deletion of the chromosomal genes was confirmed by PCR amplification and kanamycin resistance for each mutant strain. Strains used in this study are summarized in Table 4.

**Table 4:** *E. coli* strains used in this study

Name	Genotype	Reference
BW25113	F^-^, *rrnB* Δ*lacZ4787 hsdR514* Δ(*araBAD*)*567* Δ(*rhaBAD*)*568 rph-1*	(51)
JW5529	BW25113 Δ*yhbO*::*kan*, KEIO collection	(50)
JW1950	BW25113 Δ*hchA*::*kan*, KEIO collection	(50)
MC4100	*araD139* Δ(*argF*-*lac*)*U169 rpsL150 relA1 flbB5301 deoC1 ptsF25 rbsR*	(57)
CU141	MC4100/F’*lacI*^q^*lacZYA*^+^	(58)
YH2949	CU141, Δ*yhbO*	This study
YH2951	CU141, Δ*hchA*	This study
YH2970	CU141, Δ*yhbO* Δ*hchA*	This study

### Detection of CML and MG-H1 modifications

WT *yhbO*, mutant *yhbO*, or the empty plasmid pTWV228 (vector control) were transformed into the Δ*yhbO* Δ*hchA* double deletion strain, and a single colony was cultured in M9 minimal medium [47.5 mM Na_2_HPO_4_, 22 mM KH_2_PO_4_, 18.7 mM NH_4_Cl, 0.4% glucose, 0.1 mM CaCl_2_, 2 mM MgSO_4_] at 37 °C overnight. The pre-culture was diluted 20-fold and cultured for 1 h. To induce YhbO expression, bacteria were incubated with 1 mM IPTG and 1 mM adenosine 3′,5′-cyclic monophosphate (cAMP, Nacalai) for 3 h, followed by 2 mM GO or 0.5 mM MGO for 1 h. To prepare cell lysates, bacterial pellets were collected, and proteins were precipitated with 10% trichloroacetic acid. The pellets were washed with acetone and resuspended in SDS sample buffer containing DTT. Proteins were separated by SDS-PAGE and transferred to a PVDF membrane. The membrane was blocked with 2.5% skim-milk/TBS-T, then incubated with a primary antibody for 2 h and then a HRP-conjugated secondary antibody for 1 h. Primary antibodies used included: anti-CML (CosmoBio, 1:800), anti-MG-H_1_ (Cell Biolabs, 1:4000), anti-YhbO (homemade, 1:10,000), anti-HchA (homemade, 1:1000), and anti-MBP (New England Biolabs, 1:10,000). Proteins were detected using a Western Lighting Plus-ECL Kit and the FUSION SOLO S system (VILBER). Band intensities were quantified using ImageJ (version 1.53t).

### Assessment of *E. coli* survival in the presence of GO and MGO

The double deletion strain Δ*yhbO* Δ*hchA* expressing WT YhbO, mutant YhbO, or the empty plasmid pTWV228 (vector control) was cultured as described above. Following induction of YhbO expression with IPTG and cAMP, the cultures were grown to an A600 of 0.3. Cells were then serially diluted (10^−1^, 10^−2^, and 10^−3^) and spotted onto M9 minimal agar plates supplemented with 1 mM IPTG, 1 mM cAMP, and either 6 mM GO or 0.7 mM MGO. Cell survival was evaluated after incubation for 20 to 55 h.

### Molecular modeling and energy minimization

The initial model structures of YhbO complexed with GO, MGO, and PGO were constructed based on the ligand-free structure of *E. coli* YhbO (PDB: 1OI4) and the crystal structure of AtDJ-1d from *A. thaliana* (PDB: 4OGG) (30), which was solved in a covalently bound state with glyoxylate. First, the AtDJ-1d structure was superimposed onto the *E. coli* YhbO structure using PyMOL 3.1.0 [http://www.pymol.org/pymol (2025) by Schrödinger, L. and DeLano, W.], with the alignment based on the binding-pocket residues Ile103, Cys104, His105, and Gly106. Subsequently, GO, MGO, and PGO were individually positioned by aligning three common atoms, C1, C2, and O2, with those of the glyoxylate molecule in the AtDJ-1d structure, thereby generating the initial model structures of YhbO complexed with each substrate.

PyMOL 3.1.0 was used for structural superposition, ligand placement, and visualization, whereas energy minimization was performed using GROMACS 2025.1 (52). The protein was described with the Amber ff14SB force field (53), and the ligands were parameterized using GAFF2 (54). The RESP partial charges of GO, MGO, and PGO were derived after geometry optimization at the B3LYP/6-31+G(d,p) level and single-point calculations at the HF/6-31G(d) level using Gaussian16 [Frisch, M. J.; Trucks, G. W.; Schlegel, H. B.; *et al.* Gaussian 16 Revision C.01; Gaussian, Inc.: Wallingford, CT, (2016)], followed by RESP charge fitting with antechamber (55). Glu17 and His105 were modeled as GLH and HID, respectively. Each complex was placed in a 100 × 100 × 100 Å^3^ simulation box, solvated with the TIP3P water model (56), and neutralized with Na^+^ and Cl^−^ ions at a concentration of 0.15 M.

To preserve the catalytically relevant active-site geometry during relaxation, distance restraints were applied between the ligand and key active-site residues. In addition, harmonic position restraints were applied to the protein backbone and ligand during energy minimization. The minimized structures were used for structural analysis and were visualized using PyMOL 3.1.0 [http://www.pymol.org/pymol (2025) by Schrödinger, L. and DeLano, W.].

### Circular dichroism (CD)

CD measurements were carried out using a J-820 spectrometer (JASCO) fitted with a temperature-controlled cell holder. Far-UV CD spectra were collected over the wavelength range of 210 to 260 nm at 30 °C and 37 °C, using a quartz cuvette with a 1 mm path length. Data were recorded with a 1 nm interval and a response time of 1 s, and each spectrum represents the average of four consecutive scans. All measurements were repeated independently at least twice to confirm reproducibility, and representative datasets are shown in Figure 6*G*.

### Enrichment of CML/CEL peptides and mass spectrometry

Each bacterial strain was lysed in 6 M guanidine-HCl, 100 mM HEPES-NaOH, pH 7.5, 10 mM TCEP, and 40 mM CAA. The lysates were solubilized by heating and sonication, followed by centrifugation at 20,000×*g* for 15 min at 4 °C. The supernatants were recovered, and proteins (1 mg each) were purified by methanol-chloroform precipitation and solubilized in 150 μl of 0.1% RapiGest SF (Waters) in 50 mM triethylammonium bicarbonate. After sonication and heating, the protein solutions were digested with 10 μg of trypsin/Lys-C mix (Promega) at 37 °C overnight. The resulting peptide solutions were diluted 6-fold with HBS (50 mM HEPES-NaOH, pH 7.5, 150 mM NaCl), centrifuged, and subjected to immunoaffinity enrichment using the PTMScan Carboxymethyl/Carboxyethyl Lysine Motif kit (Cell Signaling Technology). The eluates in 0.15% TFA and 5% acetonitrile were desalted using GL-Tip SDB (GL Sciences), evaporated in a SpeedVac concentrator, and re-dissolved in 0.1% TFA and 3% acetonitrile. LC-MS/MS analysis of the resultant peptides was performed on a nanoElute 2 coupled with a timsTOF HT mass spectrometer (Bruker). The peptides were separated on a 75-μm inner diameter × 150 mm C18 reversed-phase column (Nikkyo Technos). The mobile phase consisted of 0.1% formic acid in water (solvent A) and 0.1% formic acid in acetonitrile (solvent B). Peptides were loaded onto the column at a flow rate of 0.2 μl/min starting at 3% B, which was linearly ramped to 32% B over 90 min, then raised to 95% B at 91 min, and held at that level until 101 min. The mass spectrometer was operated in parallel accumulation–serial fragmentation (PASEF) mode. The *m/z* range for both MS1 and MS2 spectra was 100 to 1700, and the ion mobility range was 0.6 to 1.6 Vs/cm^2^. The ramp time was 100 ms, with a duty cycle of 100%. Each acquisition cycle consisted of 10 PASEF MS2 scans. A polygon filter was applied in the m/z and ion mobility space to exclude low m/z, singly charged ions from precursor selection. The raw data were processed using the FragPipe (v22.0). Database searches were performed with the MSFragger (v4.1), employing the default parameters of the LFQ workflow against the UniProt *E. coli* (strain K12) database (6472 entries). Carbamidomethylation of Cys (+57.0215 Da) was set as a fixed modification. The following variable modifications were included: acetylation of the protein N-terminus (+42.0106 Da); oxidation of Met (+15.9949 Da); carboxymethylation (+58.0055 Da) or carboxyethylation (+72.0211 Da) of Lys. The resulting identifications were filtered using Philosopher with default parameters (MSBooster was disabled), and IonQuant (v1.10.27) was used for quantification with default software settings. Intensities of peptides containing CML or CEL were imported into Perseus (v2.1.3.0). Finally, missing values were imputed using the Perseus function “replace missing values from normal distribution” with the following parameters: width −0.3; down shift - 1.8; mode - separately for each column. Intensities of peptides containing CML or CEL were imported into R version 4.5.1 running in [_rstudio_2025].06.13. For each peptide with CML or CEL modifications, intensity values were extracted, and those with all-zero values across samples were removed. The intensity values were then log_2_-transformed. Missing values were imputed for each sample separately using random numbers drawn from a normal distribution with a width of 0.3 and a downshift of 1.8 relative to the mean intensity of the corresponding sample. Fold changes were calculated as the difference in mean log_2_ intensity between two groups, and statistical significance was assessed using Welch’s *t* test for each peptide. The results were also visualized as volcano plots showing log_2_ fold change *versus* –log_10_(p).

### Statistical analysis

All data are presented as means ± SD, unless otherwise indicated. Statistical significance was determined using one-way ANOVA with Dunnett’s multiple comparison test. Significance values are indicated as ∗*p* < 0.05. All statistical analyses were performed using Prism 10.

## Data availability

The mass spectrometry-based proteomics data have been deposited in the ProteomeXchange Consortium *via* the jPOST partner repository under the dataset identifier PXD069131, which is available at https://proteomecentral.proteomexchange.org/cgi/GetDataset?ID=PXD069131.

## Conflict of interest

The authors declare that they have no conflicts of interest with the contents of this article.
